# Controlling Macrophage Uptake of Gold Nanoparticles through the Design of an Effective Heterogeneous Coating

**DOI:** 10.1021/acsami.6c01799

**Published:** 2026-04-28

**Authors:** Paulo Siani, Ander Eguskiza, Giulia Frigerio, Edoardo Donadoni, Riccardo Ossanna, Martin Volk, Mathias Brust, Barbara Giovannone, Roberto Fiammengo, Cristiana Di Valentin

**Affiliations:** † Department of Materials Science, 9305 University of Milano-Bicocca, Via R. Cozzi 55, Milan, 20125, Italy; ‡ Department of Biotechnology, 19051 University of Verona, Strada Le Grazie 15, Verona, 37134, Italy; § Department of Chemistry, 4591 University of Liverpool, Liverpool L69 7ZD, U.K.

**Keywords:** gold, self-assembled monolayers, differential centrifugal sedimentation, zeta potential, molecular dynamics, coarse-grained modeling

## Abstract

Self-assembled monolayers (SAMs) on gold nanoparticles (AuNPs) enable the rational tailoring of their surface properties, a key feature with high relevance in biomedical applications. However, establishing direct links between the structure of SAMs at the molecular level and nanoparticle characteristics remains experimentally challenging, calling out for complementary computational techniques. Here, we integrate experimental techniques such as differential centrifugal sedimentation and electrophoretic light scattering with coarse-grained simulations to gain structural information for (mixed)-SAMs that present a varying degree of disorder at their hydrophilic, solvent-exposed interface. We propose a design strategy to control the disorder in the hydrophilic region of negatively charged SAMs that goes beyond the use of standard thiolated ethylene glycol oligomers. Our simulations show that the coating with the highest chain-length heterogeneity is characterized by superior hydration and a more diffuse electric double layer. These subtle differences, which become evident only by integrating experimental data with numerical predictions, have a large impact on the extent of nanoparticle internalization by macrophages. Through the elucidation of the structure–function relationship, this work provides a robust framework for the SAM composition optimization on nanoparticles.

## Graphical abstract

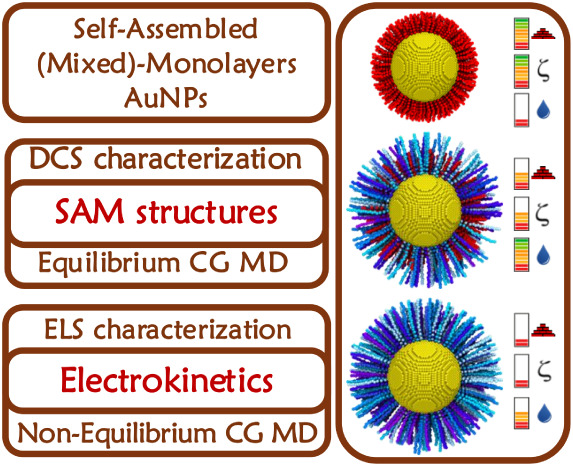

## Introduction

Gold nanoparticles (AuNPs) are widely used in biological and medical applications because of their unique properties, including their optical features in the visible range, inertness, biocompatibility, and simplicity of synthesis in different forms and dimensions.^1,2^ The functionality and dispersibility of AuNPs in various media can be controlled by the formation of self-assembled monolayers (SAMs) of thiolated ligands on their surfaces. The chemical and structural nature of these SAMs governs the interaction between the nanoparticles and their surroundings. In biological media, this interaction affects nanoparticle aggregation, protein corona formation, cellular uptake, and (intra)­cellular targeting.^3–7^ Altogether, the nature of the surface coating determines the performances of nanoparticles for biomedical applications such as drug delivery, targeted therapy, vaccine formulation, and diagnostics.^8–10^

It is generally challenging to acquire structural information at the molecular level for SAMs on AuNPs, especially concerning the order and disorder in the SAMs, despite the availability of several experimental techniques.^11^ Yet, disorder may play a central role in defining the nanoparticle surface identity. For instance, disorder resulting in enhanced hydration of SAMs on flat gold surfaces has been suggested to improve the antiadhesive properties of hydrophilic SAMs.^12^

In the case of mixed-SAMs, i.e., monolayers obtained from the coadsorption of at least two different thiols, the acquisition of structural information is even more challenging despite the fact that it is a recognized and efficient approach to modulate the physicochemical properties of nanoparticles.^11,13–15^ The synthesis of a mixed SAMs coating on AuNPs is relatively simple, but it offers the possibility to tune the surface functionality for a wide range of applications ranging from bioengineering, drug delivery, and imaging to sensing.^11,14^ In the area of biomedical applications, AuNPs coated with mixed-charge SAMs were used to selectively target lysosomes in tumor cells.^16^ Mixed SAMs of hydroxyl- and carboxyl-terminated poly­(ethylene glycol) (PEG) thiols were used to control the “stealth” properties of gold nanoparticles.^17^ Mixed SAMs of PEG-based thiols were used by some of us for the formulation of MUC1 cancer vaccines.^18,19^ An important advantage of mixed SAMs on AuNPs is their ability to improve colloidal stability and dispersion, which are critical for biodistribution and circulation time in biomedical applications. Additionally, the possibility to combine different ligands with a variety of functional groups allows customization of the nanoparticle surface. For instance, one can combine ligands that promote cell adhesion with those that enhance solution stability or biocompatibility to achieve enhanced performance. Another option is the use of amphiphilic ligands,^20,21^ where the hydrophilic portion provides stability in aqueous environments, biocompatibility, and targeting for biofunctionalization or drug delivery, whereas the hydrophobic portion enables encapsulation of hydrophobic drugs or biomolecules, promotes selective binding to lipid membranes, and facilitates targeting.

In this work, we exploit a peculiar type of mixed-SAMs on AuNPs with a core diameter ≥10 nm, namely those formed by oligomeric amphiphilic ω-carboxy-PEG alkyl thiols with a variable length of the ethylene glycol (EG) segment. We consider SAMs formed by mixtures of thiols EG_4_ (**1**) and EG_*n*_ (**2**) (Figure 1), whereby **2** itself is a mixture of homologous compounds derived from PEG with an average molecular mass of ∼600 g/mol (PEG_600_) and 7 ≤ *n* ≤ 14. Notably, thiol **1** presents only 4 EG units and is therefore appreciably shorter than any member of the oligomeric mixture EG_*n*_ **2**. Both **1** and **2** display an identical anchoring unit formed by the thiol group at the end of a C_11_-alkyl chain. By mixing different proportions of **1** and **2**, we aim at modulating the structural order of the hydrophilic, solvent-exposed region of the mixed-SAMs while keeping constant the ionizable ends. Since there is no specific physical parameter to quantify the degree of *disorder* in a coating,^22^ our work considers the degree of heterogeneity in the length distribution of the oligomeric EG segment as a proxy for *disorder*, and maximal heterogeneity is expected for an equal proportion of **1** and **2** in the SAMs.

**Figure 1:**
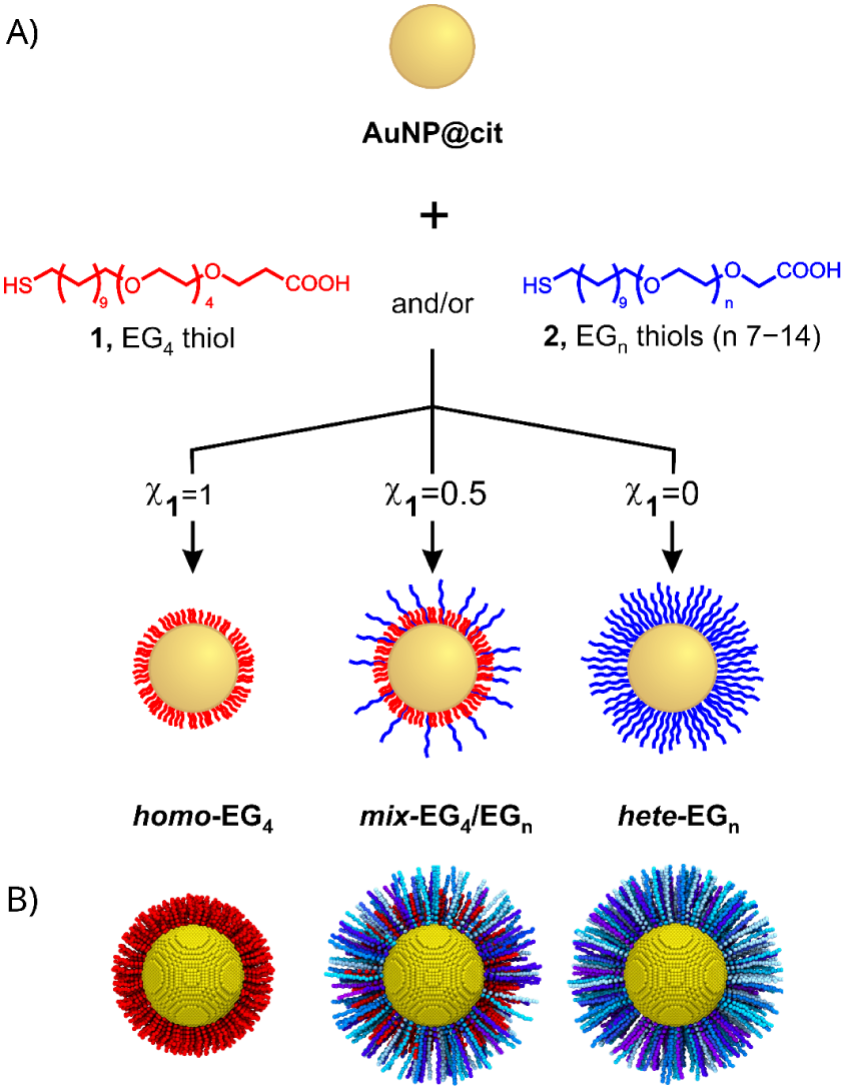
A) Schematic representation of the preparation of AuNPs coated with carboxyl-terminated ethylene glycol oligomers via the formation of mixed self-assembled monolayers (mixed-SAMs) of thiols starting from citrate-capped AuNPs (**AuNP@cit**). χ_1_ is the molar fraction of EG_4_ thiol **1** in the overall thiol mixture. B) Starting-point structures for the CG simulation of a 10 nm AuNP core coated with homogeneous EG_4_ SAMs (***homo-*** **EG_4_**), 50/50 EG_4_/EG_*n*_ SAMs (***mix-*** **EG_4_/EG_*n*_**), or heterogeneous EG_*n*_ SAMs (***hete-*** **EG_*n*_**). EG_4_ thiols are depicted in red, and EG_*n*_ thiols are shown in a blue gradient ranging from dark to light corresponding to their increasing chain lengths (7 ≤ *n* ≤ 14). Solvent molecules and the front half of the coating thiols are omitted for clarity; only one grafting density (3.0 thiols/nm^2^) is shown.

Some experimental attempts to investigate the structural features of SAMs on AuNPs have been made using nuclear magnetic resonance (NMR) especially for small AuNPs with a core diameter below 2 nm.^23,24^ Also, for very small AuNPs (gold nanoclusters with diameters <2 nm), single-crystal X-ray structures have been obtained showing the arrangement and the molecular interaction between the thiolate ligands on the Au surface.^25–27^ For larger AuNPs, with core diameters ≥10 nm, the amount of molecular details obtainable from experimental characterization methods is limited despite their relevance for biomedical applications. Atomistic simulations have emerged as suitable techniques to advance computational studies for small-sized gold nanoclusters and surface patches of large gold nanoparticles, unveiling molecular mechanisms underlying protein and AuNP associations.^28–30^ While atomistic simulations have investigated the impact of high-curvature surfaces on hydration and structural properties of homogeneous SAMs, these studies have primarily focused on small AuNPs with core diameters below 5 nm due to computational limitations.^31–33^ Approximated computational approaches, such as coarse-grained (CG) modeling and simulation, are particularly well-suited for assessing molecular-level details of SAM-coated AuNPs of larger sizes in solution (e.g., Singh et al., who compared atomistic and coarse-grained predictions against experimental evidence from STM images),^34^ as well as their cellular uptake (e.g., Chen et al., who combined *in vitro* experiments with multiscale simulations),^35^—systems for which experimental characterization alone is often limited.

We present a CG strategy to accurately model chain-length heterogeneity in mixed-SAM-coated AuNPs, enabling a detailed molecular assessment of their structural and conformational dynamic properties. To achieve this goal, we fed our simulation models with the specific SAM compositions by precisely replicating the experimental molar fraction of monodisperse thiols EG_4_ (**1**) and polydisperse EG_*n*_ (**2**) in mixed-SAMs. Building on these CG models, we present *in silico* electrophoretic experiments—implemented within the polarizable MARTINI CG framework for the first time—to numerically determine the zeta potential (z-potential) of SAM-coated AuNPs in solution. By combining numerical predictions with experimental measurements based on Differential Centrifugal Sedimentation (DCS), Electrophoretic Light Scattering (ELS), Dynamic Light Scattering (DLS), and Thermal Gravimetric Analysis (TGA), we uncover, with molecular-level details, how chain-length heterogeneity shapes (mixed)-SAM structure, thickness, ordering, hydration, and electrokinetic behavior at experimentally relevant spatial and temporal scales.

Finally, we show that the proposed nanoparticle coating design—focused on the heterogeneity of the oligomeric EG segment—has an important impact on nanoparticle internalization *in vivo* by macrophages. The internalization of PEG-coated AuNPs by macrophages has been investigated by several authors^36,37^ because of their impact in drug delivery, biological imaging, and immunomodulation. Here, by combining computational simulations with experimental data, we find that, while the standard physicochemical properties (core size, hydrodynamic diameter, polydispersity index, z-potential, and ligand grafting density) of the various AuNP formulations described in this work are not significantly different, the critical differential features of higher heterogeneity, increased hydration, and a more diffuse electric double layer remarkably reduce the extent of clearing by macrophages, as proved by specific *in vitro* experiments on a popular murine macrophage cell line.

## Results and Discussion

### Preparation and Characterization of AuNPs Coated with (Mixed)-SAMs

In our study, we aim to understand the impact of disorder on the hydrophilic interface of (mixed)-SAMs on AuNPs derived from oligomeric amphiphilic ω-carboxy-PEG alkyl thiols with variable lengths of the EG segment. We prepared a small library of (mixed)-SAMs-coated AuNPs, and we acquired experimental information, such as NP diameter, coating composition, and chain lengths, to feed computational CG simulations. We selected EG_4_ thiol **1** and EG_*n*_ thiol mixture **2** derived from PEG_600_ as the coating ligands.

In the design of our system, all used thiols (both **1** and **2**) display an identical hydrophobic anchoring unit formed by the thiol group at the end of a C_11_-alkyl chain.^37–39^ Citrate-capped AuNPs (**AuNP@cit**) were coated forming (mixed)-SAMs by coadsorption of thiol mixtures from an aqueous solution containing 20% v/v of ethanol (Figure 1). This approach was chosen to prevent the preferential adsorption of specific thiols from the mixtures because the hydrophobic contribution is kept constant throughout the thiol series, while the hydrophilic portion of all derivatives is expected to be well-solvated under these conditions. On the other hand, the variations in the length of the EG segment are expected to originate SAMs with a different degree of disorder in the hydrophilic, solvent-exposed region of the monolayer. Notably, thiol **1**, bearing only 4 EG units, is appreciably shorter than any component of the EG_*n*_ thiol mixture **2**. Therefore, blending different proportions of **1** and **2** allowed us to explore larger variations of structural order/disorder at the hydrophilic interface compared with the use of standard PEG oligomers.

After SAM formation (Figure 1A), the coated AuNPs were purified from excess thiols through a combination of ultrafiltration and gel filtration and then characterized. For ***mix-*** **EG**_**4**_ **/EG**_***n***_ AuNPs, coated using a thiol mixture with χ_1_ = 0.5 (molar fraction of EG_4_ thiol **1**), we quantified the actual amount of **1** in the mixed-SAM via ^1^H NMR after Au core dissolution with iodine. This measurement returned a value of 52% of EG_4_ thiol **1** in the coating (see Figure S3 NMR), matching well the value in the passivation solution and supporting the validity of our structural design.

We next measured the thiol grafting densities for ***homo-*** **EG**_**4**_, ***mix-*** **EG**_**4**_ **/EG**_***n***_, and ***hete-*** **EG**_***n***_ AuNPs using TGA on three independent nanoparticle batches for each formulation. The averaged values were approximately 4.0 thiols/nm^2^ across all AuNP types, with no statistically significant differences among the coatings (Table 1, individual data dispersion shown in Figure 2E), possibly caused by the relatively large data dispersion of the values. Nonetheless, a slight trend toward a lower grafting density was observed for the ***hete-*** **EG**_***n***_ SAMs, which are made of longer EG oligomers.

**Table 1:** Physicochemical Characteristics of AuNPs Coated by SAMs Determined by DLS and ELS^a^

	χ_**1**_	Averaged grafting density (thiols/nm^2^)^b^	Hydrodynamic diameter (nm)	Polydispersity index (PDI)	Zeta potential (mV)
**AuNP@cit**	—	—	15.4 ± 1.0^c^	0.10 ± 0.06^c^	–37 ± 6^d^
***homo-*** **EG**_**4**_	1	4.3 ± 0.4	22.2 ± 3.5^e^	0.22 ± 0.05^e^	–48 ± 7^e^
***mix-*** **EG**_**4**_ **/EG**_***n***_	0.5	4.0 ± 0.9	21.8 ± 4.0^e^	0.23 ± 0.08^e^	–44 ± 9^e^
***hete-*** **EG**_***n***_	0	3.6 ± 0.8	20.9 ± 1.0^f^	0.20 ± 0.09^f^	–40 ± 7^f^

a Measurements performed on samples suspended in 10 mM NaHCO_3_ at 25 °C unless otherwise specified.

b Values are means ± SD of 3 independently prepared batches.

c Averages of 8 batches ± SD measured in Milli-Q water.

d Averages of 3 batches ± SD.

e Averages of 6 batches ± SD.

f Averages of 8 batches ± SD.

**Figure 2:**
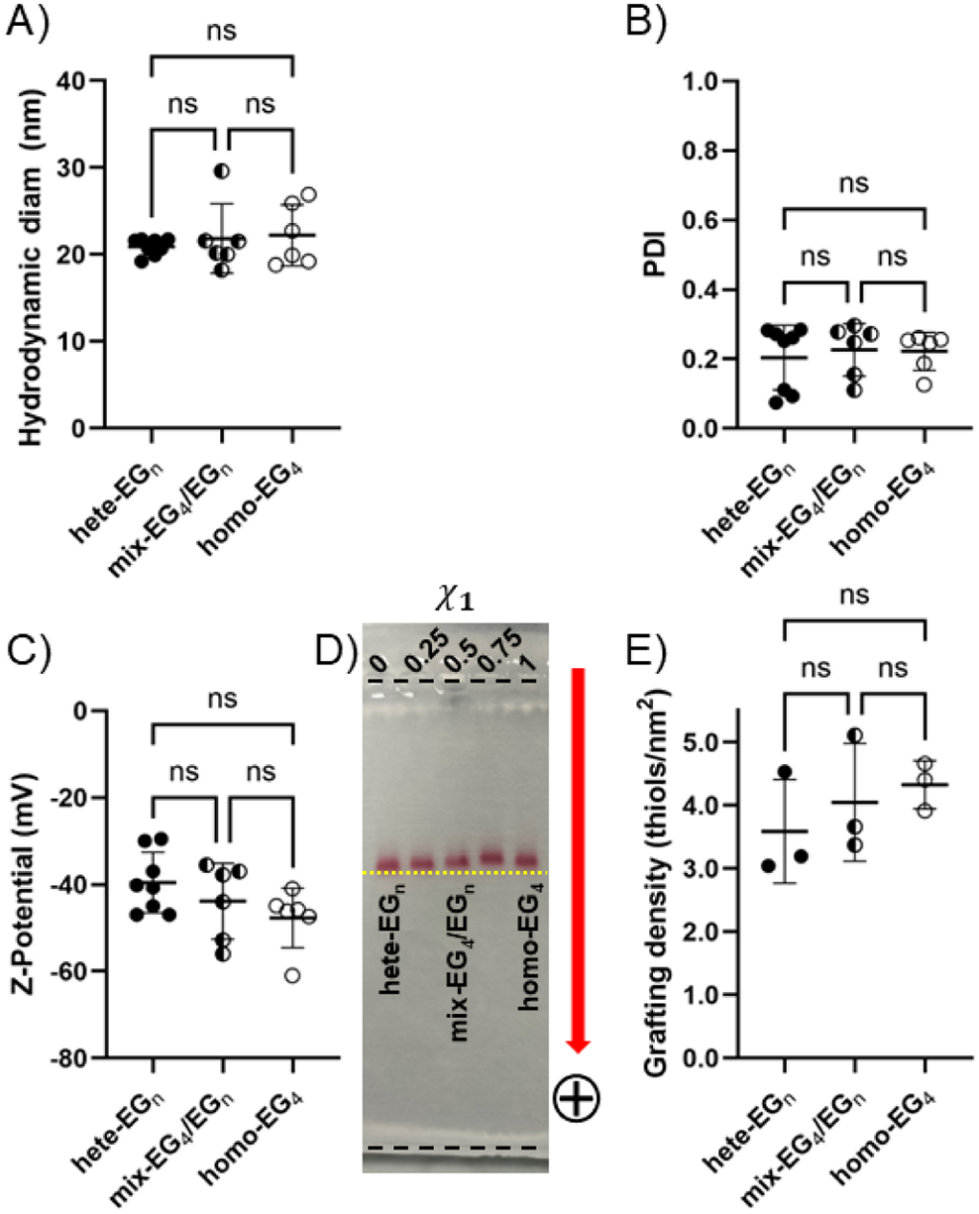
Physicochemical properties for multiple batches of SAM-coated Au nanoparticles; all graphs show measurements for individual nanoparticle batches, averages, and standard deviations; data analyzed using ordinary one-way ANOVA/Tukey-Kramer tests (A, B, and C) or ordinary one-way ANOVA/Tukey’s tests (E). A) Hydrodynamic diameter (nm); B) polydispersity index (PDI); C) z-potential (mV) in 10 mM NaHCO_3_ at 25 °C; D) gel electrophoresis on 0.6% agarose gel, running buffer 5 mM Na_2_B_4_O_7_ pH = 8.5; applied potential: 60 V; the gels show also two additional AuNP formulations with χ_1_ = 0.25 and 0.75 whose physicochemical characteristics are reported in Table S1; the yellow dotted line is a guide for the eye to appreciate the small differences in electrophoretic mobility for the 5 different formulations loaded on the gel; the black broken lines mark the position of the pockets and the end of the gel; E) grafting density measurements by thermogravimetric analysis (TGA, thiols/nm^2^), analogously to A), B), C).

These measurements are consistent with previously reported values for comparable systems: 3.9 ± 0.2 thiols/nm^2^ for HS­(CH_2_)_11_-(EG)_6_-OH determined by X-ray Photoelectron Spectroscopy (XPS),^40^ 4.3 ± 0.5 thiols/nm^2^ for HS-EG_7_-COOH measured by Inductively Coupled Plasma Mass Spectrometry (ICP-MS),^41^ and 4.2 thiols/nm^2^ for HS-EG_4_-OCH_3_ obtained by TGA.^42^ However, these literature values should be interpreted with caution, as they were derived from nanoparticles with smaller core diameters (3–6 nm) and ligands containing fewer EG units than our longer EG_*n*_ thiol **2** (PEG_600_). For larger cores (18 nm) functionalized with HS­(CH_2_)_11_-(EG)_6_-OH, a lower grafting density of 2.3–2.8 thiols/nm^2^ was determined by XPS.^40^ Similarly, a grafting density of 3.0 ± 0.2 thiols/nm^2^ was reported for 13 nm AuNPs coated with PEG_1000_-SH using both ^1^H NMR and ICP-MS.^43^

Following the results presented in the previous paragraph, we characterized a small library of (mixed)-SAM-coated AuNPs via DLS, ELS, and gel electrophoresis on agarose gels. The results of these measurements are presented in Table 1, Table S1, and Figure 2. These data were obtained from multiple independent batches, i.e., AuNPs prepared in different reactions at different times, to evaluate the variability of each nanoparticle formulation across distinct preparations. We did not observe statistically significant differences among the formulations in terms of hydrodynamic diameter, polydispersity index (PDI), or z-potential, suggesting that DLS and ELS cannot resolve subtle differences in SAM structural organization. We then selected three formulations— ***homo-*** **EG**_**4**_, ***mix-*** **EG**_**4**_ **/EG**_***n***_, and ***hete-*** **EG**_***n***_—for further investigation and computational analysis.

Although the marked increase in hydrodynamic diameter measured by DLS going from **AuNP@cit** to the variously coated AuNPs is indicative of SAM formation, this measurement provides only a semiquantitative indication of the coating thickness. This is because DLS size distributions are inherently biased toward larger particles. To obtain a more accurate assessment, we analyzed ***homo-*** **EG**_**4**_, ***mix-*** **EG**_**4**_ **/EG**_***n***_, and ***hete-*** **EG**_***n***_ nanoparticles using DCS, an independent and complementary technique. Under controlled conditions, DCS can estimate coating thickness with a precision of ∼0.1 nm. When different coatings are assembled on the same batch of **AuNP@cit**, and this initial batch is also measured, the approach enables reliable comparison of relative coating thicknesses.^44^ The size distributions obtained via DCS are shown in Figure S2, and the calculated coating thicknesses are reported in Table 2 alongside the DLS results and the TGA-derived grafting densities for the specific selected batches analyzed. From the **AuNP@cit** DCS measurements, the AuNP core diameter was calculated as 10.2 ± 0.1 nm, which matches perfectly the value obtained from TEM image analysis on 450 nanoparticles (10.2 ± 0.7 nm, Figure S1).

**Table 2:** Hydrodynamic Diameter, PDI, and Grafting Density for the Selected Batches of AuNPs Used in the DCS Measurements for Coating Thickness Determination^a^

	χ_**1**_	Hydrodynamic diameter (nm)^b^	Polydispersity index (PDI)	Grafting density (thiols/nm^2^)^c^	Coating thickness (nm)^d^
**AuNP@cit**	—	14.3 ± 0.1	0.046	—	1.0^e^
***homo-*** **EG**_**4**_	1	19.9 ± 0.2	0.126	4.7	2.2 ± 0.1
***mix-*** **EG**_**4**_ **/EG**_***n***_	0.5	21.5 ± 0.3	0.155	3.7	2.8 ± 0.1
***hete-*** **EG**_***n***_	0	21.6 ± 0.2	0.111	3.2	3.0 ± 0.1

a All samples derive from the same **AuNP@cit** batch; the diameter of the AuNP core evaluated via DCS is 10.2 ± 0.1 nm.

b Reported values are averages of 5 repeated DLS measurements ± SD for samples suspended in 10 mM NaHCO_3_ at 25 °C.

c Single TGA measurement.

d Determined via DCS. Reported values are averages of 3 repeated runs; reported uncertainties account both for the SD from repeated measurements and for some uncertainty in the assumed coating density quantified as ±0.1 g/cm^3^.

e Thickness of the adsorbed citrate layer from the literature.^45^

### Modeling AuNPs Coated with (Mixed)-SAMs Displaying Varying Chain-Length Heterogeneity

The previously described and characterized AuNPs coated by (mixed)-SAMs of amphiphilic oligo­(ethylene glycol)-derived thiols were simulated at the CG level of resolution considering their different degrees of coating heterogeneity explicitly. To model this heterogeneity, thiol ligands with variable numbers of EG units were implemented at the CG level of resolution according to the MARTINI mapping scheme. Figure 3A,B illustrates the molecular structure details of the EG_4_ and EG_*n*_ chains and their corresponding CG mapping.

**Figure 3:**
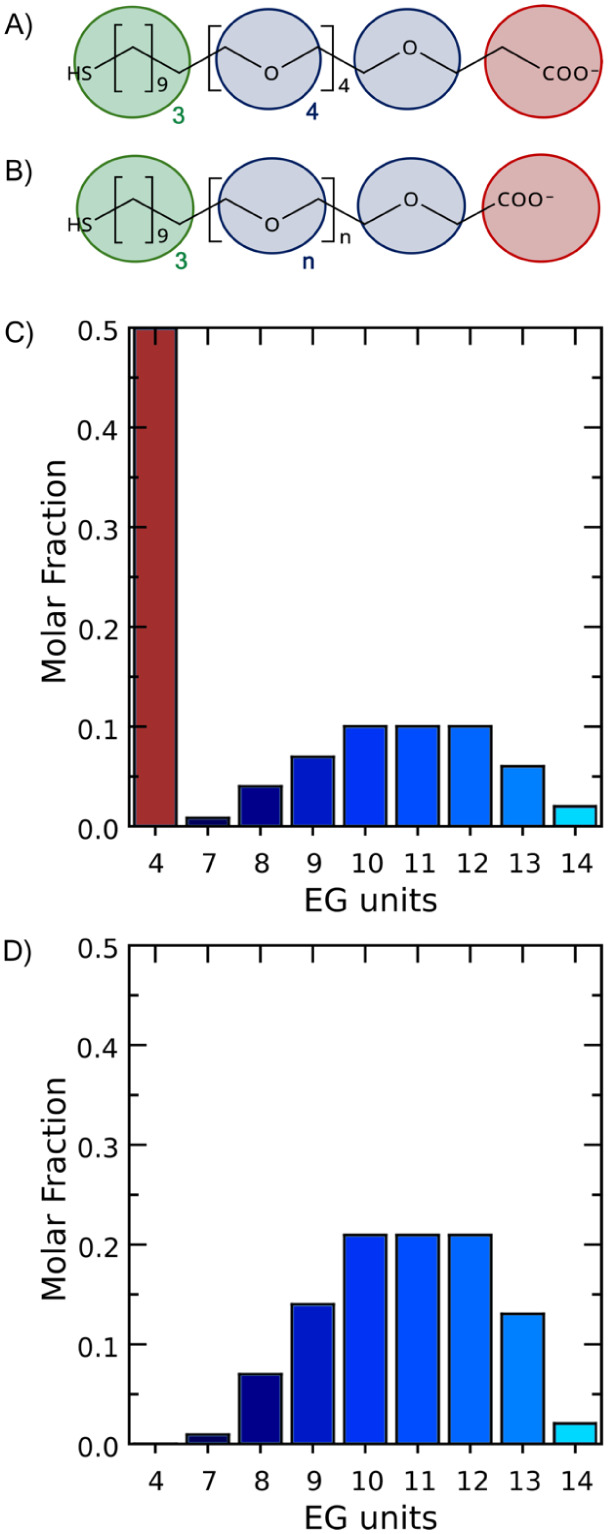
CG mapping scheme based on MARTINI building blocks, i.e., beads, for A) EG_4_ and B) EG_*n*_ chains. Color code of MARTINI bead types utilized for the CG mapping of EG_4_ and EG_*n*_ chains: HS-C_11_-alkyl moiety using the MARTINI *C1* bead type (green), with a 4-to-1 mapping (4 heavy atoms to 1 CG bead); each ethylene glycol monomeric unit using the MARTINI *EO* bead type (blue), with a 3-to-1 mapping (3 heavy atoms to 1 CG bead); and the terminal carboxylate group using the negatively charged MARTINI *Qa* bead type (red), with a 4-to-1 mapping for EG_4_ and a 3-to-1 mapping for EG_*n*_ (3 or 4 heavy atoms to 1 CG bead). Calculated molar fraction of EG_4_ and EG_*n*_ thiols in C) ***mix*** **-EG_4_/EG_*n*_** and D) ***hete*** **-EG_*n*_** coatings from MALDI-TOF mass spectrometry analysis of the EG_*n*_ thiol mixture **2** and ^1^H NMR analysis of the ***mix*** **-EG_4_/EG_*n*_** coating composition.

We built three distinct SAM-coated AuNP models (Figure 1B) feeding in the available experimental evidence, setting at 10 nm the diameter of the AuNP core, and varying the molar fraction (χ_1_) of the monodisperse component EG_4_ thiol **1** in the SAMs, namely:

- *AuNPs coated by short-chain length homogeneous SAM (* ***homo*-EG**_**4**_, χ_1_ = 1.0). Nanoparticles with homogeneous coating serve as an entry point for the simulations, being the simplest system.
- *AuNPs coated by long-chain length heterogeneous SAM (* ***hete*-EG**_***n***_, χ_1_ = 0). Nanoparticles with heterogeneous coating composed of thiols from EG_*n*_ thiol mixture **2** presenting EG_*n*_ chains of varying lengths (7 ≤ *n* ≤ 14). The relative amount of each component in the coating is set at the value measured experimentally by MALDI-TOF mass spectrometry of mixture **2** in Figure 3D. This model has the purpose of revealing the structural properties of a mixed-SAM obtainable from a standard oligomeric mixture (PEG_600_) of amphiphilic thiols.
- *AuNPs coated by short- and long-chain length heterogeneous mixed-SAM* (***mix-*** **EG**_**4**_ **/EG**_***n***_, χ_1_ = 0.50). Nanoparticles with heterogeneous coating with χ_1_ = 0.5 and the remaining 50% made of thiol mixture **2**. In such a model, it is possible to consider an even larger degree of heterogeneity of EG chain-length distribution compared to a standard oligomeric mixture.

As done in a previous study of similar systems,^46^ we carefully selected the most suitable MARTINI mapping and building blocks, i.e., beads, to closely represent the volume and expected chemical behavior in the various components of our systems. To better reproduce solvation effects, we adopted the polarizable MARTINI framework,^47^ which overcomes the oversimplified electrostatics of the standard framework^48^ by incorporating a more realistic electrostatic treatment through the inclusion of polarization effects. Detailed information on the MARTINI force field (FF) parameters used to model the alkyl-EG chains, AuNP core, solvent, and ions, including the utilized bead types, intermolecular interaction matrix, and intramolecular parameters, is reported in the Computational Methods section.

For each AuNP model, four separate CG simulations were performed, uniformly covering the AuNP surface with the appropriate number of thiols corresponding to grafting densities of 2.0, 3.0, 4.0, and 5.0 thiols/nm^2^. These values were selected to encompass the range of experimentally determined grafting densities (Figure 2E) and to elucidate how the grafting density influences the SAM structure, from loosely to more densely packed monolayers. Exploring this range of grafting densities is further justified by the difficulty of determining the exact thiol grafting density on nanoparticle surfaces with high precision using experimental techniques; substantial variability is often observed among values reported by different laboratories and different analytical methods, even for the same system.^43^

### Predictions of SAM Thickness: Computational Results vs Experimental Measurements

We compared the SAM thickness predictions derived from CG simulations with those measured experimentally using DCS. The goal of this comparison was to assess the accuracy and robustness of the CG models in reproducing experimental data. In previous work,^49^ we assessed the accuracy of similar CG models against their atomistic counterpart, where we see that the SAM thickness is overestimated with the former models by approximately 9% and 19% at high and low grafting density regimes for the ***homo*** **-EG**_**4**_ coating, respectively (see Reliability and Validation of the MARTINI CG Models, SI). SAM thickness, *G*_coat_, was derived from the cumulative radial distribution function of all beads constituting the SAM coatings, calculated according to eq 1:

$$G_{\mathrm{coat}}\left(r\right)=\frac{1}{N_{\mathrm{coat}}}\int_{0}^{r}\overset{N_{\mathrm{coat}}}{\underset{i=1}{\sum }}\langle \delta_{D}\left(r_{i}-r^{\prime }\right)\rangle {dr}^{\prime }$$ 1

In this expression, δ_D_ is the Dirac delta function and *N*_coat_ is the total number of beads in the SAM, where *r*_i_ denotes the radial position of each individual bead, while *r*' is the integration variable used to accumulate these positions from the particle center outward. Following a similar approach used in a previous study,^46^ the distribution was integrated to find two radial cutoff distances, *r_5%_* and *r_95%_*, relative to the AuNP surface. The first cutoff *r_5%_* corresponds to the radial distance from the AuNP surface that contains 5% of the coating beads, whereas the second cutoff, *r_95%_*, defines the distance containing 95% of the coating beads. Thus, *G*_coat_, calculated as *r_95%_−* *r_5%_,* represents the radial interval within which 90% of the coating beads are found. This parameter was used to evaluate the ability of the CG models to predict the monolayer thickness measured experimentally by DCS, which serves as the reference data set in this study. Figure 4 presents the SAM thickness predictions obtained from CG simulations at grafting densities of 2.0, 3.0, 4.0, and 5.0 thiols/nm^2^ (using the definition in eq 1) alongside the corresponding experimental DCS measurements from the previous section.

**Figure 4:**
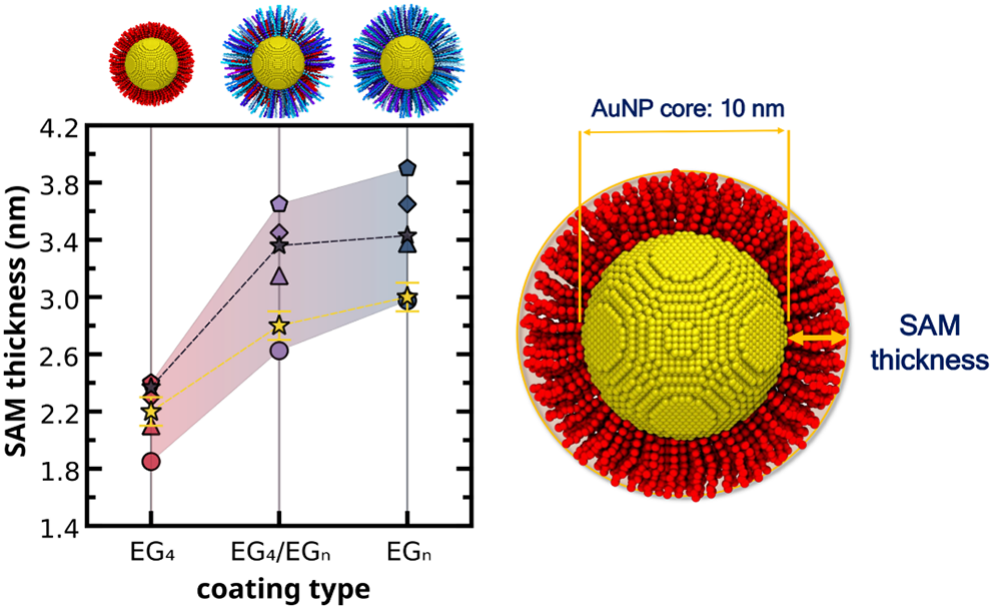
Computational predictions and experimental values of the thickness of self-assembled monolayers on ***homo*** **-EG_4_** (pink), ***mix*** **-EG_4_/EG_*n*_** (purple), and ***hete*** **-EG_*n*_** AuNPs (blue) for varying grafting densities. Symbol code: experimental DCS measurements of SAM thickness (yellow stars) and CG-extrapolated SAM thickness at the experimental TGA grafting densities (gray stars), CG predictions at grafting density of 2.0 thiols/nm^2^ (circles), 3.0 thiols/nm^2^ (triangles), 4.0 thiols/nm^2^ (diamonds), and 5.0 thiols/nm^2^ (pentagons). The error bars for the experimental values account both for the SD from repeated measurements and for some uncertainty in the assumed coating density quantified as ±0.1 g/cm^3^. Reported computational values are averages ± SD taken over the last 1 μs of the production phase. For clarity, the nanoparticle images on this figure only show AuNPs with a grafting density of 3.0 thiols/nm^2^.

The computational predictions show a substantial increase in SAM thickness from ***homo*** **-EG**_**4**_ to ***mix*** **-EG**_**4**_ **/EG**_***n***_ and ***hete*** **-EG**_***n***_ coatings, consistent with the increasing lengths of the capping ligands. It is worth noting that the CG simulations were run at a fixed grafting density for all AuNP coatings (ranging from 2.0 to 5.0 thiols/nm^2^), whereas the experimental DCS data (**yellow stars**) suggest potential differences in grafting density, with decreasing values for ligands of increasing chain length. TGA measurements for the specific AuNP batches used in the DCS experiments (Table 2) also indicate a decrease in grafting density from ***homo*** **-EG**_**4**_ to ***mix*** **-EG**_**4**_ **/EG**_***n***_ and ***hete*** **-EG**_***n***_ coatings; however, these differences were not statistically significant across *n* = 3 independent batches (Figure 2E and Table 1) as discussed previously for averaged grafting density values.

For ***homo*** **-EG**_**4**_, the CG predictions at grafting densities of 3.0 and 4.0 thiols/nm^2^ provide lower and upper bounds for the SAM thickness that bracket the experimental DCS-derived values (Figure 4). As the grafting density increases, the difference in SAM thickness between ***mix*** **-EG**_**4**_ **/EG**_***n***_ and ***hete*** **-EG**_***n***_ coatings becomes progressively smaller, reflecting the reduced free volume per chain and the increased packing efficiency of the ligands on the nanoparticle surface. However, for both ***mix*** **-EG**_**4**_ **/EG**_***n***_ and ***hete*** **-EG**_***n***_ systems, high grafting densities of 4.0 and 5.0 thiols/nm^2^ largely overestimate the SAM thickness relative to the experimental DCS data (yellow stars).

If the grafting density values estimated from TGA measurements (4.7, 3.7, and 3.2 thiol/nm^2^ for the AuNP batches) are adopted to the ***homo*** **-EG**_**4**_, ***mix*** **-EG**_**4**_ **/EG**_***n***_, and **hete-EG**_***n***_ coatings, respectively, the SAM thickness values (gray stars in Figure 4), linearly extrapolated from the CG data set, are found to overestimate the corresponding SAM thicknesses by approximately 8%, 20%, and 14%. For the ***homo*** **-EG**_**4**_ coating, the 8% overestimation of the SAM thickness is consistent with the corresponding ∼9% estimated from comparison with atomistic simulation predictions, as mentioned above.^49^ Interestingly, despite this systematic overestimation in the CG simulations, the experimentally observed trend in SAM thickness is respected, as highlighted by the analogy between the dashed lines connecting the experimental (yellow stars) and extrapolated (gray stars) values in Figure 4.

The dependence of SAM thickness on grafting density is also spotted for each individual system (see values along single vertical lines in Figure 4). For the ***homo*** **-EG**_**4**_ coating, variations in grafting density from 2.0 to 5.0 thiols/nm^2^ have only a minor effect on the SAM thickness, whereas the same variation produces a remarkably more pronounced impact on the SAM thicknesses of ***mix*** **-EG**_**4**_ **/EG**_***n***_ and ***hete*-EG**_***n***_ coatings. This behavior is further corroborated by analysis of the radius of gyration (RoG): while the RoG of EG_4_ chains remains practically constant across the different grafting densities, a monotonic increase is observed for the EG_17_ chains in the ***mix*** **-EG**_**4**_ **/EG**_***n***_ and ***hete*-EG**_***n***_ coatings (see Figure S6 in Supporting Computational Data, SI) from 2.0 to 5.0 thiols/nm^2^. These higher RoG values indicate more stretched chains upon increasing the grafting density, reflecting their tighter packing on the AuNP surface.

In line with this trend, the computational results further show that increasing the EG_*n*_ fraction also leads to a denser distribution of the ligand’s distal beads interfacing the solution. This accounts for the consistently thicker SAM layers in the ***hete-*EG**_***n***_ systems (Figure 4), which in turn create a more tightly packed outer region of the SAM layer that is likely to expel solvent more efficiently than the ***mix-*EG_4_/EG**_***n***_ coating—an effect that will be quantitatively investigated in the following section.

### Effect of Chain-Length Heterogeneity on SAMs Hydration

We quantified the amount of water as a fraction of the total SAM volume for all three AuNP models. To quantify the water content within the coating, the computationally predicted SAM thickness for each AuNP formulation at different grafting densities (2.0–5.0 thiols/nm^2^) was used as the radial boundary to define the monolayer volume. The water volume fraction, ϕ_w_, was then calculated using eq 2:

2 $$\phi_{w}=\frac{V_{wat}}{V_{\mathrm{total}}}=\frac{V_{wat}}{V_{wat}+V_{pol}}$$

where *V*_wat_ and *V*_pol_ represent the volumes occupied by solvent beads and polymer chains, respectively, within the defined radial cutoff. The total monolayer volume is then given by *V*_tot_ = *V*_wat_ + *V*_pol_. The solvent contribution, *V*_wat_, was estimated by summing the volumes of all solvent beads whose positions fall within the SAM region, defined as the radial domain extending from the AuNP surface up to the selected radial cutoff distance, using the Lennard-Jones bead size defined for the polarizable water in the MARTINI force field. The remaining volume within this region was attributed to the polymer phase (*V*_pol_). This approach implicitly assumes a complete partitioning of the monolayer volume between solvent and polymer components. Figure 5 shows an overall reduction in the water fraction within the SAM as the grafting density increases from 2.0 to 5.0 thiols/nm^2^, regardless of the coating type.

**Figure 5:**
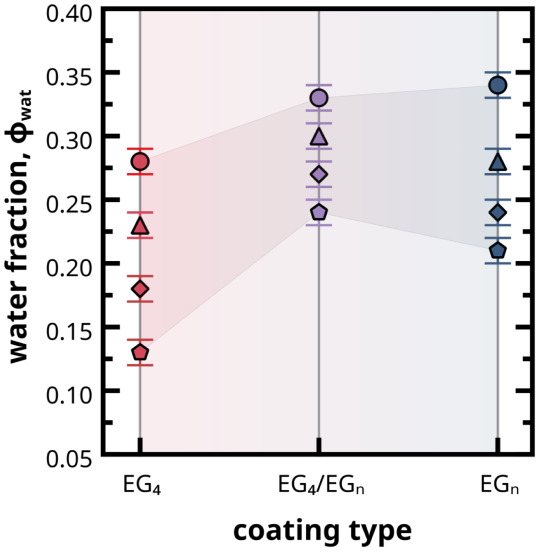
Fraction of water molecules within SAM coatings of ***homo*** **-EG_4_** (pink), ***mix***-**EG_4_/EG_*n*_** (purple), and ***hete***-**EG_*n*_** (blue) AuNPs at varying grafting densities. Symbol code: CG predictions of water volume fraction within SAM at grafting density of 2.0 thiols/nm^2^ (circles), 3.0 thiols/nm^2^ (triangles), 4.0 thiols/nm^2^ (diamonds), and 5.0 thiols/nm^2^ (pentagons). Reported values are averages ± SD taken over the last 1 μs of the production phase.

This reduction was primarily driven by the increase in the coating material at a higher grafting density, resulting in more tightly packed ligand chains on the AuNP surface. This tight packing effectively excluded water molecules from this region, especially for the ***homo*** **-EG**_**4**_ coating as one would expect, since it is constituted of thiols with the shortest hydrophilic segment. Previous studies of the spatial distribution of water content have found similar behavior in homogeneous SAMs, in which a transition from highly hydrated to dehydrated zones within SAMs is found moving from bulk water to the NP core.^50^

There is a significant increase in water content for the ***mix*** **-EG**_**4**_ **/EG**_***n***_ coating compared to ***homo*** **-EG**_**4**_. This increase is even more pronounced at higher grafting densities (Figure 5). This is not surprising considering that replacing half of the short EG_4_ chains with longer EG_*n*_ chains results in a less dense and more loosely packed outer layer of the SAM with a lower relative content of EG, favoring water uptake. Interestingly, for the ***hete*-EG**_***n***_ coating, where the SAM is composed solely of EG_*n*_ chains with lower heterogeneity compared to the EG chains in the ***mix*-EG**_**4**_ **/EG**_***n***_ coating, the water fraction within the SAM consistently decreases at grafting densities equal to or higher than 3.0 thiols/nm^2^, while remaining essentially constant at 2.0 thiols/nm^2^.

At low grafting density (2.0 thiols/nm^2^), the ***hete*-EG**_***n***_ and ***mix*-EG**_**4**_ **/EG**_***n***_ coatings exhibit similar water fractions because the surface is sparsely populated in both cases, leaving sufficient space for water beads to penetrate in between polymer chains similarly. As the grafting density increases (3.0–5.0 thiols/nm^2^), the ***hete*-EG**_***n***_ brush becomes more crowded, with longer chains locally blocking access to shorter chains and creating regions of high polymer density. This patchy, irregular brush structure reduces water accessibility, leading to a lower water fraction compared to ***mix*-EG**_**4**_ **/EG**_***n***_, which in turn allows water beads to occupy interstitial spaces more easily.

This result shows that coatings with higher heterogeneity in the length distribution of the oligomeric EG segment, i.e., more disordered coatings, are characterized by a significantly higher water content within the SAM. Interestingly, our mixed-SAMs design, with higher heterogeneity compared to what is reachable with standard PEG oligomers (where chain heterogeneity is dictated by PEG synthesis), allowed us to reach higher hydration values.^51^

The water content of the different coatings can also be estimated from the experimental data. The ligand grafting density measured by TGA, together with the AuNP core size determined by DCS and the density of PEG, yielded an estimate of the volume of the thiol chains, *V*_pol_, which can be compared to the total coating volume, *V*_tot_, as estimated from the DCS results. This yields a water volume fraction, ϕ_wat_, of 0.05, 0.28, and 0.30, respectively, for the batches of ***homo*** **-EG**_**4**_, ***mix*** **-EG**_**4**_ **/EG**_***n***_, and ***hete*** **-EG**_***n***_ AuNPs used in the DCS measurements, which had grafting densities of 4.7, 3.7, and 3.2 thiols/nm^2^, respectively (Table 2). The experimental values of water content for ***homo*** **-EG**_**4**_, ***mix*** **-EG**_**4**_ **/EG**_***n***_, and ***hete*** **-EG**_***n***_ coatings align very well with the simulation results obtained for a grafting density of about 5.0, 3.0, and 2.5 thiols/nm^2^, respectively, which confirms the qualitative predictive capability of the implemented CG framework also with respect to solvation effects. A much lower solvation was found experimentally for AuNPs with a ***homo*** **-EG**_**4**_ coating, although the exact value depends on the grafting density used for the estimate.^46^ Irrespective of the exact value, the lower solvation of the ***homo*** **-EG**_**4**_ coating indicates that it is easier to achieve higher grafting densities for shorter and more homogeneous ligands; the tight packing required for such a high grafting density precludes the uptake of a significant amount of water.

### Ordering in SAM Coatings: The Effect of Chain-Length Heterogeneity

To understand how ligand chain length and coating composition affect ligand conformation and the overall ordering within SAMs, we introduce the *order parameter* ⟨*S*⟩, in analogy to the order parameter definition commonly adopted in biophysical studies of phase transitions in lipid membranes,^52^ investigation of molecular ordering in liquid crystals,^53^ and computational studies of nanoparticle–polymer composites.^31^ According to eq 3,

3 $$\left\langle S\right\rangle =\left\langle \frac{3}{2}\cos^{2}\left(\theta_{n}\right)-\frac{1}{2}\right\rangle$$

θ_n_ is the angle formed between the radial principal axis given by the unit vector connecting the geometric center of the AuNP core to the grafting point of each thiol ligand on the AuNP surface and the segment vector, *n⃗*, connecting two consecutive CG beads in the thiol chain. ⟨*S*⟩ = 1 means perfect alignment, and therefore, an anisotropic orientational behavior of the *n⃗* vector relative to the principal axis vector, whereas ⟨*S*⟩ = 0 corresponds to random alignment and therefore an isotropic orientational behavior of *n⃗* with respect to the principal axis vector.

Figure 6 shows ⟨*S*⟩ values for the thiol ligands constituting the ***homo*** **-EG**_**4**_, ***mix*** **-EG**_**4**_ **/EG**_***n***_ and ***hete*** **-EG**_***n***_ coatings. ⟨*S*⟩ values were calculated over all *n⃗* vectors connecting consecutive pairs of identical CG beads in EG_4_ chains (***homo*** **-EG**_**4**_ and ***mix*** **-EG**_**4**_ **/EG**_***n***_ coatings in Figure 6A), both EG_4_ and EG_*n*_ chains (***mix*** **-EG**_**4**_ **/EG**_***n***_ in Figure 6B), and EG_*n*_ chains (***hete*** **-EG**_***n***_ in Figure 6B), over the last 1 μs of CG production trajectories. In this discussion, we show only the ⟨*S*⟩ values for the *n⃗* vectors, oriented radially outward from the AuNP surface, connecting pairs of consecutive CG beads making up the intermediate and outer region of the SAM, namely EG-EG and EG-COO^–^ bonds, respectively. Additional ⟨*S*⟩ values for the remaining *n⃗* vectors constituting the connecting pairs of CG beads mapping the C_11_-alkyl moiety can be found in Tables S3 and S4.

**Figure 6:**
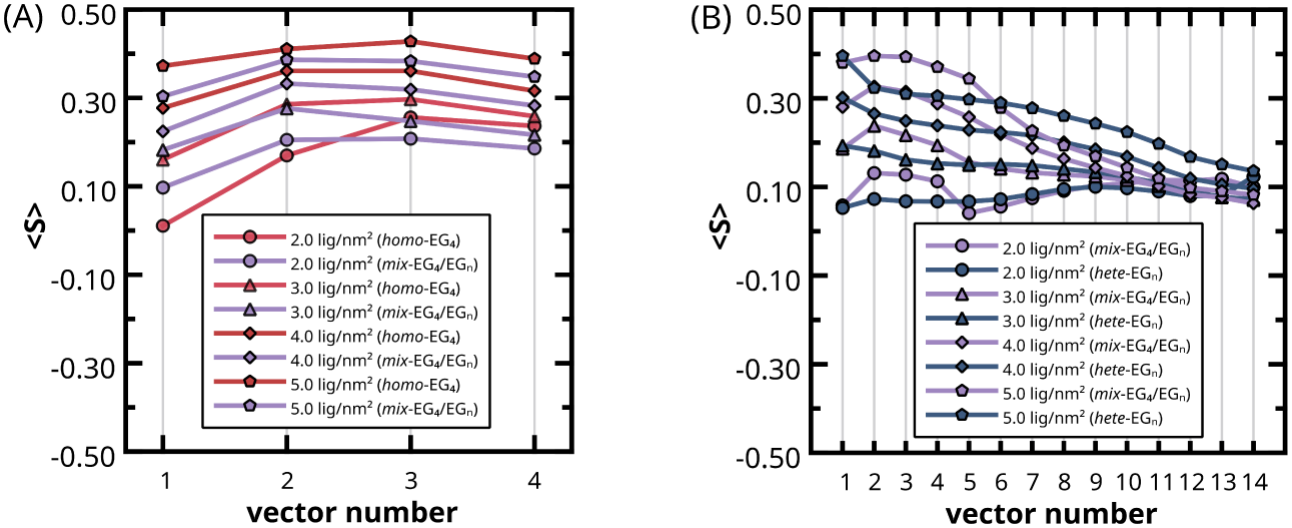
Order parameter of bead–bead bond vectors of: A) EG_4_ chains in ***homo*** **-EG_4_** (pink) and ***mix*** **-EG_4_/EG_*n*_** (purple) coatings. B) EG_4_/EG_*n*_ chains (purple) in ***mix*** **-EG_4_/EG_*n*_** and EG_*n*_ chains (blue) in ***hete*** **-EG_*n*_** coatings. Symbols: grafting density of 2.0 thiols/nm^2^ (circles), 3.0 thiols/nm^2^ (triangles), 4.0 thiols/nm^2^ (diamonds), and 5.0 thiols/nm^2^ (pentagons). Reported values are averages taken over the last 1 μs of the production phase and complementary averages and their standard deviation values can be found in Tables S3 and S4.

From a direct comparison of the order parameter ⟨*S*⟩ for the EG_4_ chains of thiol **1** for ***homo*** **-EG**_**4**_ and ***mix*** **-EG**_**4**_ **/EG**_***n***_ AuNPs in Figure 6A, we see that the EG_4_ chains in ***mix*** **-EG**_**4**_ **/EG**_***n***_ behave very similarly to the EG_4_ chains in ***homo*** **-EG**_**4**_, showing that the presence of 50% thiol **2** with longer EG_*n*_ chains has substantially no influence on the EG_4_ chain ordering. The higher the grafting density, the higher the ordering of EG_4_ chains in ***homo*** **-EG**_**4**_ and ***mix*** **-EG**_**4**_ **/EG**_***n***_ brushes.

Conversely, the presence of 50% EG_4_ chains in ***mix*** **-EG**_**4**_ **/EG**_***n***_ coatings has an impact on the ordering of the longer EG_*n*_ chains at all simulated grafting density regimes, as shown in Figure 6B. The deviation in structural order between ***mix*-EG**_**4**_ **/EG**_***n***_ and ***hete*-EG**_***n***_ coatings at low grafting densities (2–3 thiols/nm^2^) is confined to the inner EG units (up to *n* = 4–5). On the other hand, at higher grafting densities (4–5 thiols/nm^2^), the distal region of ***hete*-EG**_***n***_ coatings consistently shows higher structural order than the corresponding region in ***mix*-EG**_**4**_ **/EG**_***n***_ coatings. In fact, for ***mix*** **-EG**_**4**_ **/EG**_***n***_ coatings, but not for ***hete*** **-EG**_***n***_, the order parameter ⟨*S*⟩ of EG_*n*_ chains initially increases and remains higher than in the ***hete*** **-EG**_***n***_ up to the fourth EG unit, i.e., until the length of the EG_4_ chains is reached. At higher grafting densities (4.0 or 5.0 thiols/nm^2^), this effect extends to the fifth EG unit. This behavior reflects the presence of shorter, more ordered EG_4_ chains in the inner region of the ***mix*** **-EG**_**4**_ **/EG**_***n***_ coating, which, due to their restricted mobility, promote increased ordering of neighboring EG_*n*_ chains. In contrast, EG_*n*_ chains in ***hete*** **-EG**_***n***_ coatings, which lack this inner-region constraint, exhibit lower ordering in the same region and present a rather monotonic decrease of ⟨*S*⟩ (or practically constant values for lower grafting densities) moving toward the end of the chains converging to similar values irrespectively of the coating morphology and grafting density regime adopted (Figure 6B, blue vs purple curves). Beyond the fifth EG unit, ⟨*S*⟩ values for EG_*n*_ chains in ***mix*** **-EG**_**4**_ **/EG**_***n***_ and ***hete*** **-EG**_***n***_ coatings are very similar and low for grafting densities of 2.0–3.0 thiols/nm^2^, while they are higher and clearly different, with lower ⟨*S*⟩ values for EG_*n*_ chains in ***mix*** **-EG**_**4**_ **/EG**_***n***_, for grafting densities of 4.0–5.0 thiols/nm^2^. In other words, starting from a grafting density of 4.0 thiols/nm^2^, i.e., if the grafting density is high enough and comparable to that determined experimentally for our systems, the EG_*n*_ chains in ***mix*** **-EG**_**4**_ **/EG**_***n***_ coatings are more disordered than in ***hete*** **-EG**_***n***_. Regardless of the grafting density, ⟨*S*⟩ converges to low values toward the solution, indicating greater orientational freedom for the terminal EG units. These values align well with the results of previous computational studies of homogeneous SAM coatings on highly curved surfaces of ultrasmall Au cores.^31^

Thus, based on ⟨*S*⟩ computation, our CG model shows that ***mix*** **-EG**_**4**_ **/EG**_***n***_ AuNPs present increased order in the intermediate hydrophilic region of the coating SAM compared to ***hete*** **-EG**_***n***_ AuNPs but at the same time increased *disorder* beyond this region toward the solvent-exposed interface, if the grafting density is high enough and comparable to that determined experimentally for our systems. Such a detailed view of the coating morphology cannot be obtained directly from experimental data, underscoring the importance of computational simulations in achieving this level of structural resolution. We speculate that the differences in coating structural order described above in terms of ⟨*S*⟩ values may result in differences in SAM compliance when the nanoparticles interact with biological interfaces such as in the formation of protein corona or in the interaction with cell membranes, e.g., during nanoparticle internalization.

### Zeta Potential of SAM-Coated Gold Nanoparticles: A Comparison of NEMD Predictions and ELS Measurements

An important advantage of coating AuNPs with SAMs of thiolated ligands is the possibility of tailoring the overall electric charge of the nanoparticle by a judicious choice of the chemical characteristics of the coating ligands (e.g., their terminal group). The z-potential, an electrokinetic property of nanomaterials, is fundamental for controlling nanoparticle dispersion and stability in aqueous solutions and is often measured through ELS. Despite its significance, the role of z-potential in understanding nanoparticle interactions with biological media—such as drug delivery, protein corona formation, and cell internalization^54,55^ —has been overlooked but is now gathering attention in the design of advanced nanomedical devices.^56^

In this section, we assess the predictive power of CG simulations to quantify the electrokinetic properties of the implemented SAM-coated AuNP models, specifically electrophoretic mobility and the corresponding z-potential, using experimental ELS data as reference. We performed Nonequilibrium Molecular Dynamics (NEMD) simulations to calculate the electrophoretic (EP) velocity of the SAM-coated AuNPs in the direction of an applied external electric field and their EP mobility for the last 1 μs of NEMD production phase according to the mathematical relationship given by eq 4:

4 $$\mu_{x}^{EP}=\frac{v_{x}}{E_{x}}$$

where $\mu_{x}^{EP}$ is the average EP mobility, estimated from the limiting drag velocity (*υ*_*x*_) of the center-of-mass of the SAM-coated AuNPs, and *E_x_* is the electric field applied in the *x*-direction of the simulation box. The z-potential values, ζ, are then derived from the Helmholtz–Smoluchowski relation among the EP mobility, viscosity, and dielectric constant of the medium, given by eq 5:

5 $$\zeta =\frac{\eta \mu_{x}^{EP}}{\varepsilon_{0}\varepsilon_{r}}$$

in which ε_0_ is the vacuum permittivity, ε_r_ is the relative dielectric constant, and η is the viscosity of the medium. For the z-potential predictions in Figure 7, we used the average viscosity (3.0 × 10^–4^ Pa · s) obtained from the Equilibrium Molecular Dynamics (EMD) simulation replicas via the Einstein relation in eq 8 (please see Estimation of Shear Viscosity, Computational Methods), along with the dielectric constant (75.6) of the refined polarizable MARTINI water model as reported in its original parametrization.^47^

**Figure 7:**
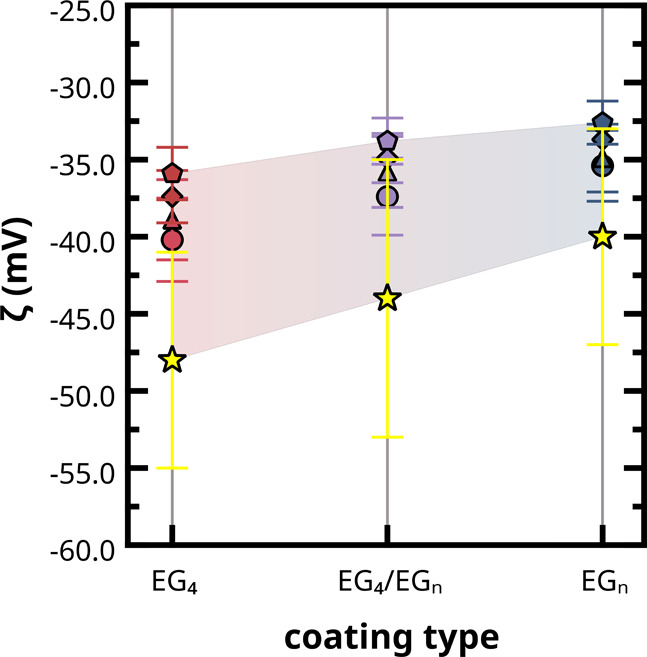
z-Potential predictions for ***homo*** **-EG_4_** (pink), ***mix*** **-EG_4_/EG_*n*_** (purple), and ***hete*** **-EG_*n*_** (blue) AuNPs. Symbol code: ELS experimental data (stars), and NEMD predictions at grafting densities of 2.0 thiols/nm^2^ (circles), 3.0 thiols/nm^2^ (triangles), 4.0 thiols/nm^2^ (diamonds), and 5.0 thiols/nm^2^ (pentagons) at 10 mM NaCl. Reported values are averages ± SD taken over the last 1 μs of the production phase and can be also found in Table S5. Complementary z-potential values predicted at 150 mM NaCl can be found in Table S6.

In the NEMD simulations, the CG beads representing the terminal carboxylic acid groups were all considered to be deprotonated. Hence, the total net charge of the SAM-coated AuNPs is given by the sum of these negatively charged beads, which in turn matches the number of thiol ligands grafted on the AuNP surface. To maintain electroneutrality, an equivalent number of Na^+^ counterions was added, followed by additional Na^+^ and Cl^–^ ions to set the ionic strength of the medium at 10 mM of NaCl and reproduce the experimental ELS ionic strength conditions (for further details on the NEMD simulation protocol, refer to the Computational Methods).

The z-potential values for the different AuNP formulations across the grafting density regimes from 1.0 to 5.0 thiols/nm^2^ are presented in Figure 7. The NEMD CG predictions align qualitatively well with the trends observed for the experimental ELS-derived data. Interestingly, as χ_1_ decreases in the SAM going from ***homo*** **-EG**_**4**_ to ***mix*** **-EG**_**4**_ **/EG**_***n***_, and finally reaching ***hete*** **-EG**_***n***_, there is a gradual, nearly linear increase of z-potential toward less negative values. This trend was not statistically significant for repeated z-potential measurements on independently prepared nanoparticle batches (Figure 2C). Considering that the total negative net charge is identical across all considered CG AuNPs (at the same fixed grafting density values), we attribute the observed differences in z-potential values to the formation of dissimilar electric double-layer structures across the three different AuNP formulations.

To evaluate the electric double layer structure and radial extent to which the ion distribution screens the surface charge on SAM-coated AuNPs, we estimated the cumulative radial charge screening profiles, κ­(*r*), for all three SAM compositions: ***homo*** **-EG**_**4**_, ***mix*** **-EG**_**4**_ **/EG**_***n***_, and ***hete*** **-EG**_***n***_, with the ionic strength of the medium set at 10 mM of NaCl. The κ­(*r*) is calculated as defined in eq 6:

$$\kappa \left(r\right)=\int_{0}^{r}\left[\rho_{q^{-}}\left(r^{\prime }\right)+\rho_{q^{+}}\left(r^{\prime }\right)\right]{dr}^{\prime }$$ 6

where $\rho_{q^{-}}$ stands for the radial charge distribution of negatively charged beads, namely COO^–^ groups and Cl^–^ ions, and $\rho_{q^{+}}$ stands for the charge distribution of positively charged beads, namely Na^+^ ions. From the radial charge screening profiles in Figure 8 (and their respective radial charge distribution profiles in Figure S7), it is possible to appreciate that ***homo*** **-EG**_**4**_ presents a tighter arrangement of Na^+^ counterions and the formation of a more compact electric double layer (Figure 8, red curves) setting the slipping plane closer to the AuNP surface. On the other hand, for ***mix*** **-EG**_**4**_ **/EG**_***n***_ and ***hete*** **-EG** ***n***, the presence of increasing amounts of thiol mixture **2** in the SAM with longer EG_*n*_ chains spreads out the negative charge over a larger radial distance, with counterions and co-ions now forming a more diffuse electric double layer (Figure 8, blue and violet curves) and shifting the slipping plane at a farther distance from the AuNP surface compared to ***homo*** **-EG**_**4**_. Interestingly, both experimental and simulation-derived z-potential data suggest that ***mix*** **-EG**_**4**_ **/EG**_***n***_ presents an electric double layer diffused at a similar extent to that of ***hete*** **-EG**_***n***_, as seen by their charge screening profiles in Figure 8. Increasing the grafting density shifts the slipping plane further into the solution and, in the case of ***mix*-EG4/EG**_***n***_ also promotes a more structured negatively charged inner region within the SAM, as reflected by a transition from unimodal to bimodal profiles (Figure 8).

**Figure 8:**
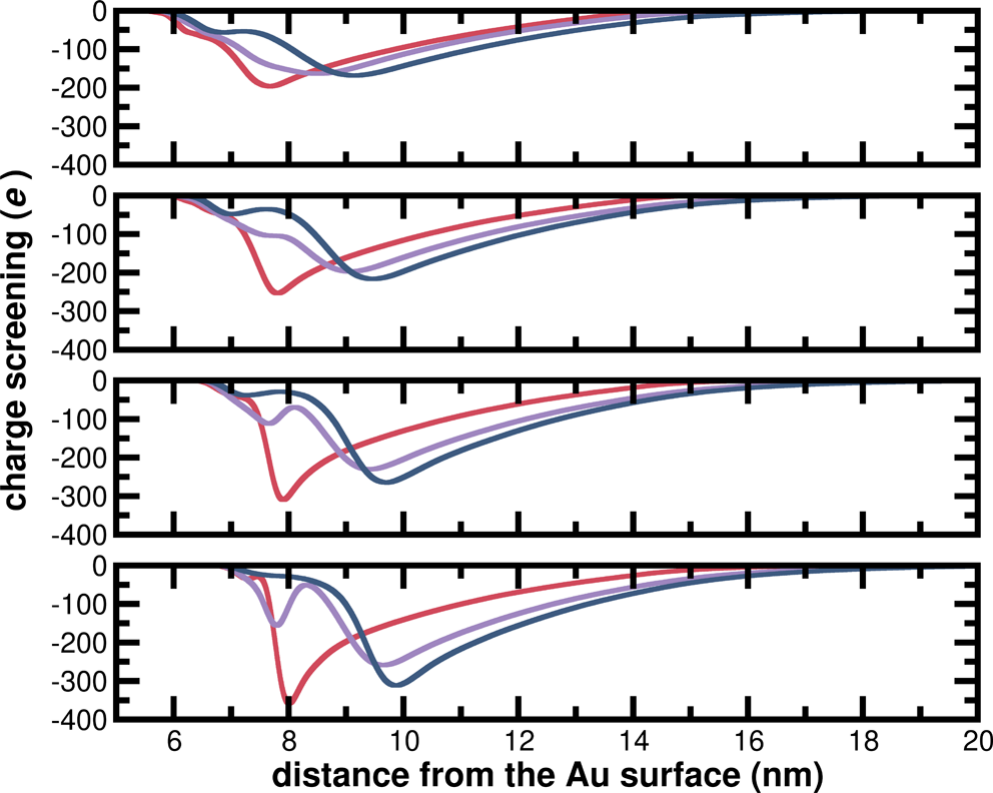
Charge screening profiles for ***homo*** **-EG_4_** (pink), ***mix*** **-EG_4_/EG_*n*_** (purple), and ***hete*** **-EG_*n*_** (blue) AuNPs at: A) 2.0 thiols/nm^2^, B) 3.0 thiols/nm^2^, C) 4.0 thiols/nm^2^, and D) 5.0 thiols/nm^2^. Average charge screening profiles are calculated over the last 1 μs of the production phase. Complementary charge screening profiles calculated at 150 mM NaCl can be found in Figure S5.

From the comparison discussed in this paragraph, we conclude that ***homo*** **-EG**_**4**_ AuNPs present a more compact electric double layer and a slipping plane closer to the AuNP surface, therefore resulting in a more negative z-potential. In contrast, the more diffuse electric double layer possessed by ***mix*-EG**_**4**_ **/EG**_***n***_ and ***hete*** **-EG**_***n***_ AuNPs results in a slipping plane farther away from the AuNP surface and a less negative z-potential.

Moreover, z-potential values predicted *in silico* using electrophoretic NEMD CG simulations are systematically less negative than experimental values from ELS (up to 10–15 mV for ***homo*** **-EG**_**4**_ AuNPs), regardless of the grafting density considered. This discrepancy is primarily attributed to the enhanced diffusivity—and consequently, lower shear viscosity—of the polarizable MARTINI water model relative to the experimental values (Figure 7). These deviations stem from intrinsic limitations of most CG approaches, particularly the ones lacking an explicit hydrogen bonding description, that are otherwise preserved in fully atomistic water models. The lower viscosity inherent to the refined polarizable MARTINI water model likely results in faster ion electrophoretic mobility; however, this effect is partially offset in eq 5 by the reduced shear viscosity constant, thereby shifting the computed z-potential toward less negative values than the experimental reference data. While this is a well-known limitation of most CG models and there is still room for further FF refinements, the predicted z-potential values fall within the experimental uncertainty range across all systems studied.

To gain a qualitative understanding of how chain heterogeneity influences the charge density distribution around the AuNPs, we generated electrostatic potential maps to visualize the spatial distribution of charge on ***homo*** **-EG**_**4**_, ***mix*** **-EG**_**4**_ **/EG**_***n***_, and ***hete*** **-EG**_***n***_ AuNPs. A qualitative analysis of Figure 9 reveals that the ***homo*** **-EG**_**4**_ coating exhibits a smoother surface with a higher negative charge density (magenta spots in Figure 9A). On the other hand, increasing the EG_*n*_ content resulted in SAMs displaying a rougher surface with negative charges spread out in larger volumes, yielding a lower negative charge density (green spots, Figures 9B and 9C). Moreover, an even rougher SAM surface is observable for ***mix*** **-EG**_**4**_ **/EG**_***n***_ compared to ***hete*** **-EG**_***n***_, consistent with the presence of both EG_4_ and EG_*n*_ chains, demonstrating again that the solvent-exposed interface for these two systems is appreciably different. It is also worth noting that all CG models assume complete deprotonation of the terminal carboxyl groups, representing the limiting case of a coating with the highest possible negative charge density. Previous theoretical studies have shown that the p*K*_a_ of carboxyl groups on ligands grafted onto AuNPs may shift toward higher values, particularly for larger AuNPs such as those used in our study and under low ionic strength conditions.^57^ This p*K*_a_ shift is expected to affect the protonation state of the terminal carboxyl groups in our coatings, a factor that was not accounted for in the current CG modeling.

**Figure 9:**
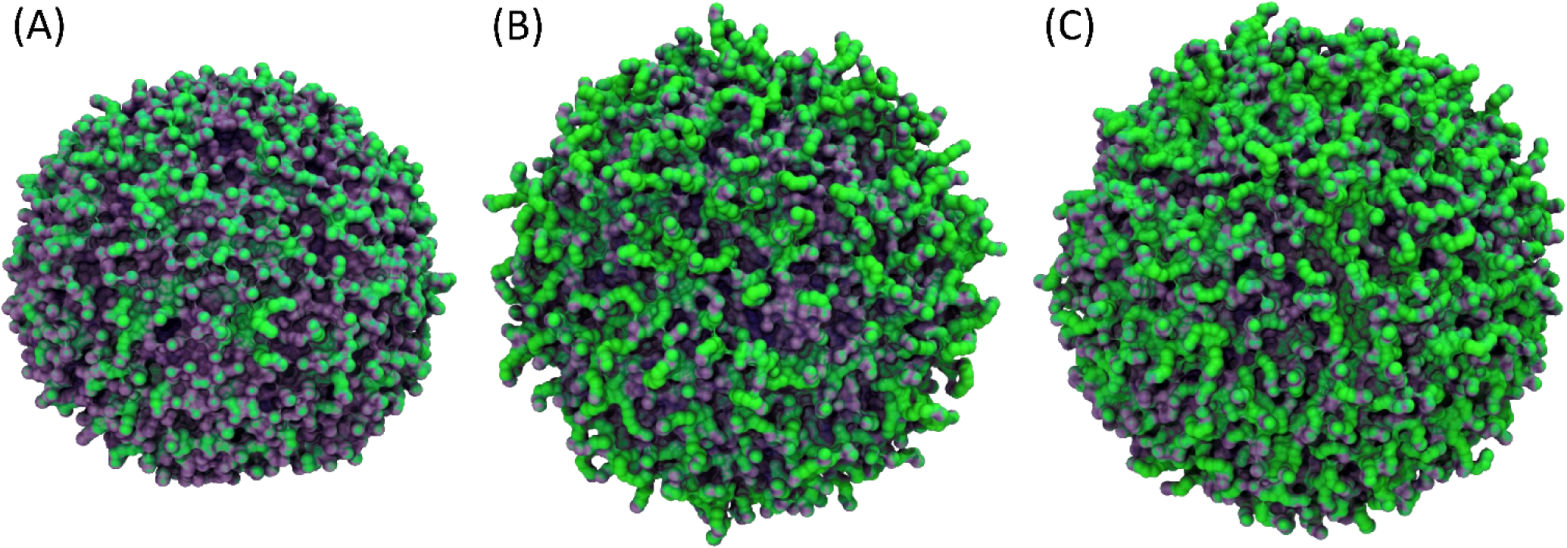
Electrostatic potential maps for (A) ***homo*** **-EG_4_**, (B) ***mix*** **-EG_4_/EG_*n*_**, and (C) ***hete*** **-EG_*n*_** AuNPs at a grafting density of 3.0 thiols/nm^2^. The magenta-to-green color scale depicts the SAM regions transitioning from a negative to neutral surface charge potential.

Considering all of the intrinsic approximations in the implemented electrophoretic *in silico* experiments, we confirmed the reliability of NEMD simulations within this CG framework in predicting the correct trend (not absolute values) of experimental z-potential values, in fair agreement with their corresponding ELS measurements.

### Structural Features of SAM Coatings Modulate Nanoparticle Uptake by Cells

With our CG simulations, we have shown that ***homo*** **-EG**_**4**_, ***mix*** **-EG**_**4**_ **/EG**_***n***_, and ***hete*** **-EG**_***n***_ AuNPs present distinct coating morphologies with notable differences in SAM order, water content, and charge density. Considering SAM order and hydration, ***mix*** **-EG**_**4**_ **/EG**_***n***_ represents a unique case compared to single-component SAMs. With these premises, we set out to investigate whether these differences would impact phagocyte uptake by using the murine macrophage cell line RAW264.7. The clearance of PEGylated AuNPs by macrophages has been investigated by several authors because of their relevance in assessing the applicability of nanoparticle-based formulations in the biomedical field.^36,37,58^

In this experiment, we tested the complete small library of mixed-SAM-coated AuNPs including formulations ***mix-25*** **EG**_**4**_ **/PEG** and ***mix-75*** **EG**_**4**_ **/PEG** (χ_1_ = 0.25 and 0.75, respectively, Table S1). Prior to cell experiments, each nanoparticle formulation was diluted to a concentration of 6 nM in a mixture containing 60% v/v fetal bovine serum (FBS) and 40% phosphate-buffered saline (PBS) at pH 7.4 and incubated overnight at room temperature to allow protein corona equilibration. These mixtures were then used to prepare the complete cell culture medium (Dulbecco’s Modified Eagle’s Medium (DMEM) + 10% FBS) supplemented with 1 nM AuNPs. Macrophages were incubated in such AuNP-containing complete media for 3 h before being washed to remove excess nanoparticles that were not internalized. The number of internalized nanoparticles per cell was determined by gold quantification via ICP-MS on mineralized cell pellets dividing by the number of cells (Figure 10).

**Figure 10:**
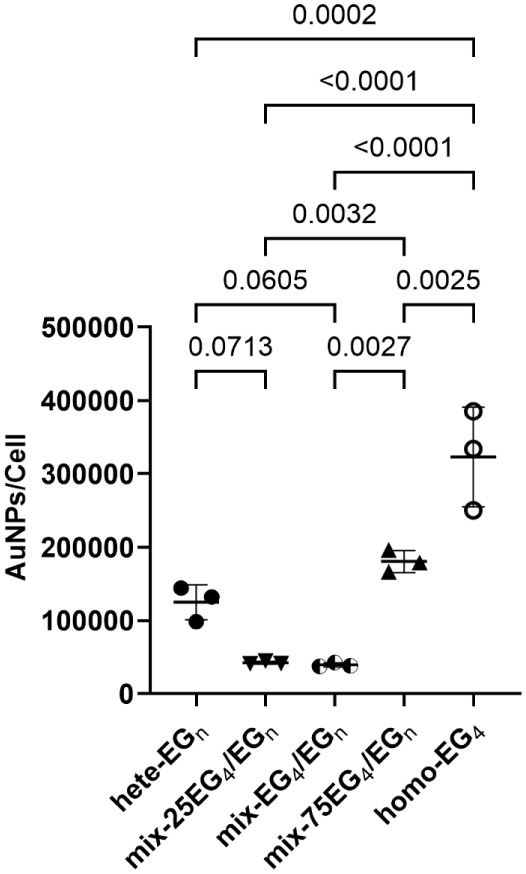
Nanoparticle uptake by the murine macrophage cell line RAW264.7 after 3 h incubation with 1.0 nM of the different SAM-coated AuNPs in complete cell culture medium, evaluated via inductively coupled plasma mass spectrometry (ICP-MS). Data are presented as scatter plots showing means ± SD; one-way ANOVA/Tukey’s post hoc tests. Multiplicity adjusted *P*-values are shown only for *P* < 0.1.

Our measurements show that effective nanoparticle clearance by macrophages at 3 h follows a nonmonotonic trend (with respect to SAM composition) with minimal internalization for ***mix*** **-EG**_**4**_ **/EG**_***n***_ and ***mix-25*** **EG**_**4**_ **/PEG** formulations. Notably, ***mix*** **-EG**_**4**_ **/EG**_***n***_ AuNPs are phagocytosed approximately 8 times less efficiently than ***homo*** **-EG**_**4**_ AuNPs and still approximately 3 times less than ***hete*** **-EG**_***n***_ AuNPs. These results show that the structural architecture of the coating SAMs, and in particular their disorder/hydration, likely has a significant effect on nanoparticle opsonization and, therefore, on the recognition by macrophages. Although very striking, we acknowledge that this result has some limitations including a) the analysis of nanoparticles with a fixed size of around 20 nm hydrodynamic diameter, yet relevant, e.g., in the field of vaccine formulations; b) the analysis at a fixed nanoparticle concentration for a period of 3 h because the study focuses on the clearance process; and c) the use of FBS as a protein source in the context of a murine cell line. These limitations should be addressed by additional experimental work on nanoparticle formulations for pharmaceutical use using primary cells, species-relevant protein sources, e.g., human serum or plasma, and extended time and concentration intervals to assess dose-related efficacy and any possible toxicity-related side effects.

As noted in previous works, nanoparticles in biological media may transition into a “triple system” comprising the gold core, the surface coating, and an evolving protein corona.^59^ This adsorbed protein layer can penetrate the polymer brush, potentially altering the nanoparticle’s charge distribution and biological identity. However, PEG antifouling performance depends on its ability to resist protein adsorption and suppress protein corona formation, a property arising from a hydrated interface of tightly bound, hydrogen-bonded water molecules, with PEG ligands serving primarily as hydrogen-bond acceptors.

It has been experimentally demonstrated that approximately one water molecule is tightly bound to each EG unit using low-field nuclear magnetic resonance (LF-NMR) and differential scanning calorimetry (DSC) measurements in PEG/water mixtures.^60^ Notably, similar behavior—about one water molecule per EG unit—was observed in previous simulations by some of us of PEGylated titania nanoparticles using a classical force field approach.^61^ Experimental and computational findings have further supported^62–64^ the importance of interfacial water in regulating protein adsorption onto polymer brush surfaces, showing that strong water–polymer interactions effectively suppress protein adsorption through the formation of a stable hydration layer. Combined 3D frequency-modulation atomic force microscopy and MD simulations have further revealed that this hydration layer generates water-mediated repulsive forces that hinder protein interactions and preserve nanoparticle’s biological identity.^65^

Among all formulation parameters of SAM AuNPs, the degree of SAM’s hydration—verified herein by the fraction of water over polymer content (Figure 5) across the different formulations—best correlates with the experimental macrophage uptake observed in Figure 10. The ***mix*** **-EG**_**4**_ **/EG**_***n***_ coating exhibits the highest SAM’s hydration—which at high grafting density regimes reflects a strongly bound solvent population—and correspondingly shows the lowest macrophage uptake in Figure 10, suggesting impaired protein corona formation. The ***hete*** **-EG**_***n***_ system displays intermediate SAM’s hydration and macrophage uptake, whereas ***homo*** **-EG**_**4**_ shows the lowest coating hydration and the highest macrophage uptake. Overall, these findings indicate a direct correlation between the degree of hydration of different SAM coating morphologies—quantified here as the water fraction within the SAM coating in Figure 5—and macrophage clearance, implying the extent of protein corona formation. Therefore, the degree of SAM’s hydration stands out as the most relevant parameter correlating with the observed experimental trends in macrophage uptake.

## Conclusion

This study combines CG simulations and experimental techniques to describe the structural features and the electrokinetic behavior of (mixed)-SAM-coated AuNPs in aqueous solution. The ***homo*** **-EG**_**4**_ AuNPs coated by short-chain-length thiol **1** present a compact SAM with higher ligand ordering that largely excludes water. The solvent-exposed interface appears smooth and with higher negative charge density and more negative z-potential compared to the other two AuNP formulations. In contrast, ***mix*** **-EG**_**4**_ **/EG**_***n***_ and ***hete*** **-EG**_***n***_ AuNPs coated by mixed-SAMs including long-chain-length thiols **2** present reduced ordering in the SAM’s outer region, a more diffuse electric double layer resulting in reduced surface charge density, and less negative z-potential. While most of the characteristics of ***mix*** **-EG**_**4**_ **/EG**_***n***_ and ***hete*** **-EG**_***n***_ seem to be dominated by the presence of EG_*n*_ thiols **2**, the CG modeling allows to spot subtle differences between these two AuNP formulations which could not be clearly identified experimentally. In particular, the water content at the high grafting density regime (3.0–5.0 thiols/nm^2^, enclosing the experimental value) is higher for ***mix*** **-EG**_**4**_ **/EG**_***n***_ than for ***hete*** **-EG**_***n***_. Our results suggest that SAM’s hydration correlates with macrophage uptake, where higher coating hydration is associated with reduced immune recognition and clearance, likely through modulation of protein corona formation.

Likewise, the ordering of the coating thiols is enhanced by the presence of EG_4_ thiols **1** in ***mix*** **-EG**_**4**_ **/EG**_***n***_ compared to ***hete*** **-EG**_***n***_, independent of the grafting density. The CG simulations also spotted a trend in the grafting density, which did not reach statistical significance in the experimental determination over several AuNP batches. We finally show that ***mix*** **-EG**_**4**_ **/EG**_***n***_ AuNPs can escape the murine macrophage (cell line RAW264.7) approximately 8 times more efficiently than ***homo*** **-EG**_**4**_ AuNPs and still approximately 3 times more efficiently than ***hete*** **-EG**_***n***_ AuNPs.

Overall, these results demonstrate that numerical simulations, integrated with experiments, offer an effective and robust approach for shedding light into the structural, conformational, and electrokinetic behavior of mixed SAMs-coated AuNPs with core diameters ≥10 nm, especially when the differences are subtle and difficult to reveal and quantify through experimental physicochemical measurements. However, they become evident and decisive when the AuNPs are evaluated for macrophage uptake assays *in vitro*—a key determinant of nanoparticle clearance and biodistribution—which are expected to translate into prolonged *in vivo* circulation time.

## Methods

### Experimental Methods

#### Preparation of AuNPs

Citrate-coated AuNPs (**AuNP@cit**) with a diameter of 10.2 ± 0.7 nm (see Figure S1) were synthesized according to a literature procedure.^66^ AuNPs were coated (passivated) by the formation of SAMs of thiols on their surface as previously reported^67^ with small variations described here. Mixed SAMs were prepared using mixtures of EG_4_ thiol (**1**) and polydisperse EG_*n*_ thiol **2**, with a molar fraction of **1** (χ_1_) as indicated in the main text. Briefly, stock solutions of thiols in EtOH (5 mM) were freshly prepared and directly used. Final concentrations in the passivation reaction were ∼100 nM AuNPs, 25 mM NaHCO_3_, 1 mM thiols (total concentration in case of thiol mixtures), and therefore, the reaction contained 20% v/v of EtOH. The passivation reactions were performed in closed glass vials, protected from light, at r.t., and with gentle magnetic stirring (200–300 rpm) for 96 h. AuNPs were purified using Vivaspin Turbo 4 Ultrafiltration Units MWCO 100 kDa (washing with 1 × 4 mL 25 mM NaHCO_3_, 1 × 4 mL 2:8 v/v EtOH/50 mM NaHCO_3_ and 2 × 4 mL 50 mM NaHCO_3_, and 2 × 4 mL H_2_O). AuNPs were then taken up in H_2_O and further purified by gel filtration on NAP-10 columns preconditioned with H_2_O. AuNP eluates were collected following the manufacturer’s instructions. The final concentration of AuNPs in the purified samples varied in the interval 200–400 nM depending on the volume of the passivation reactions (4–8 mL).

#### Differential Centrifugal Sedimentation (DCS)

The size distribution of AuNP samples was measured using DCS in a DC24000 disc centrifuge (CPS Instruments Inc.). The disc, rotating at 24,000 rpm, was filled by successive addition of sucrose solutions (8–24 wt % sucrose in Milli-Q H_2_O), and the instrument was calibrated using 0.377 μm poly­(vinyl chloride) particles (Analytik Ltd.). Each sample was analyzed three times to verify data reproducibility. As previously described,^44^ the DCS software underestimates the size of AuNPs coated with an organic layer (see Figure S2) because it ignores the lower density of the coating layer, but this can be quantitatively corrected for. The measurements performed for citrate-capped AuNPs (**AuNP@cit**) allow a highly precise estimate of the gold core diameter (independently from TEM measurements) which turned out to be 10.2 ± 0.1 nm, assuming a citrate layer density of 1.4 g/cm^3^ and a citrate layer thickness of 1 nm.^44^ The measurements for ***homo-*** **EG**_**4**_, ***mix-*** **EG**_**4**_ **/EG**_***n***_, and ***hete*** **-EG**_***n***_ yielded the thickness of the respective coating layer with an accuracy of 0.1 nm; here, a density of 1.12 g/cm^3^ was used for both EG_4_ thiol **1** and EG_*n*_ thiol mixture **2**.^68^ It is important to note that DCS can yield such a precise measurement of the coating thickness for differently coated AuNPs if they all derive from a single **AuNP@cit** batch. Hydrodynamic diameter, PDI, grafting density, and coating layer thickness for the specific batches analyzed via DCS are reported in Table 2.

#### Dynamic Light Scattering (DLS) and Zeta Potential Measurements

The hydrodynamic diameter (mean z-average diameter) and the polydispersity index (PDI) of the AuNPs were determined by DLS via cumulant analysis of the time autocorrelation function using a Litesizer DLS 500 instrument (Anton Paar). AuNPs were suspended in 10 mM NaHCO_3_ in water at 25 °C. Zeta potentials were measured by electrophoretic light scattering (ELS) in the same conditions. The results for multiple batches of nanoparticles independently prepared are reported in Table 1. Statistical analysis of the results for ***hete-*** **PEG** (*n* = 8), ***mix-*** **EG**_**4**_ **/PEG** (*n* = 6), and ***homo-*** **EG**_**4**_ (*n* = 6) coated AuNPs was performed via ordinary one-way ANOVA and posthoc Tukey-Kramer test.

### Computational Methods

#### MD Simulation Details

All EMD and NEMD simulations utilized the polarizable MARTINI water model refined for Particle Mesh Ewald (PME) electrostatics,^47^ with a distance of 0.14 nm between the central W particle and the oppositely charged WP and WM dummy beads (*q* = ±0.457 *e*) constrained utilizing the LINCS algorithm.^69,70^ Consistently, we adopted the long-range solver smooth Particle Mesh Ewald (PME method) with a grid spacing of 0.12 nm and a short-range cutoff of 1.1 nm for the calculation of electrostatic interactions.^71^ To ensure the compromise of a realistic dielectric behavior in hydrophilic and hydrophobic regions, the background dielectric constant was set to ϵ_r_ = 2.5 as recommended for the polarizable MARTINI water model refined for PME electrostatics.^47^ A potential-shift cutoff scheme was utilized for Lennard–Jones (12–6) interactions in which the energy was smoothly shifted to zero within a cutoff distance of 1.1 nm. A minimization phase using the steepest descent algorithm was carried out with a number of 50,000 steps to minimize forces and the potential energy of the system, followed by an equilibration phase 200 ns long in which the systems were thermalized and pressurized at 303 K and 1 atm in the NPT ensemble utilizing the V-rescale thermostat (τ_B_ = 1.0 ps) and the isotropic Berendsen barostat (τ_P_ = 5.0 ps and κ = 4.5 × 10^–5^ bar^–1^).^72^ The production phase of EMD simulations explored 1.0 μs of the phase space in the NPT ensemble at 303 K at 1 atm, by coupling the system to the isotropic Parrinello-Rahman barostat (τ_P_ = 12.0 ps and κ = 4.5 × 10^–5^ bar^–1^)^73,74^ and to the velocity rescaling for controlling temperature as given in Bussi et al.^75^ Newton’s equations of motion were integrated in time using a time step of 20 fs. The production phase of NEMD simulations explored 1.0 μs of the phase space, using as a starting point the equilibrated structure taken from the last snapshot obtained after the 1.0 μs long EMD production run.

#### MD Simulation Protocols

Both EMD and NEMD simulations were performed using the open-source GROMACS (version 2021.3) code^76^ and the interaction matrix associated with the MARTINI v2.2refPol force field.^47,48,77^ The polymer-coated AuNPs were inserted in the center of a cubic simulation box, and the empty space was filled with water beads described by the polarizable MARTINI water model refined for PME electrostatics. A minimal bulk-water buffer of 3 nm was ensured in all system models. In the EMD simulations, an appropriate number of positively charged Na^+^ counterions was added to ensure electroneutrality of the system. In the NEMD simulations, positively charged Na^+^ counterions were added the same way as in EMD to ensure electroneutrality, adding further Na^+^ and Cl^–^ to set the ionic strength at either 10 mM or 150 mM. The electrophoretic (EP) flow was induced by an external electric field of 0.2 V/nm applied in the *x*-direction of the systems, as done in a previous study by some of us.^78^ The EP velocity of the center-of-mass of the AuNPs was collected on-the-fly and then averaged out over the last 1 μs of the production phase. The average EP velocities were then used as input in eq 4 for deriving the EP mobility and then the corresponding zeta potential values using eq 5.

#### Modeling of the 10 nm Gold Nanoparticle Core

The structure and topology for the 10 nm AuNP core was built from FCC lattices with a bulk density of 19.3 g/cm^3^ generated using the NanoModeler source code.^46^ Based on the total volume of the AuNP core, the total mass was distributed over all the available core beads to match the corresponding weight of its atomistic counterpart. We adopted a 1-to-1 mapping to represent the Au atoms, where their corresponding CG beads were modeled as purely hydrophobic moieties using the hydrophobic *C5* MARTINI building block held together by an elastic network with a force constant of 32,500 kJ mol^–1^ nm^–2^.

#### Modeling of EG_4_ and EG_*n*_ Chains

For the CG modeling of the hydrophobic HS-C_11_-alkyl portion of EG chains, we utilized a *4-to-1* mapping scheme represented by three *C1* MARTINI building blocks, each mapping four heavy atoms. Given the dominant hydrophobic contribution of the C_11_-alkyl segment, the polar contribution of the single sulfur atom at the point of attachment to the AuNP surface was disregarded. For the CG modeling of the hydrophilic EG portion, we utilized a *3-to-1* mapping scheme represented by the *EO* MARTINI building block, with each mapping three heavy atoms in each EG monomeric unit. For the CG modeling of the hydrophilic carboxylate terminal group, we utilized the *Qa* MARTINI building block for mapping three or four heavy atoms in the EG_4_ or EG_*n*_ chains, respectively. The nonbonded interaction matrix and bonded parameters for bonds, angles, and dihedrals were taken from Franco-Ulloa et al.,^46^ which are based on previous parametrization by Rossi et al.^79,80^ In all cases, we ensured that the exact molecular weight for the EG_4_ and EG_*n*_ chains at the CG resolution level matched exactly their atomistic counterparts. The attachment of coating ligands through covalent bonds to their anchoring sites on the 10 nm AuNP core surface was generated using the NanoModeler source code.^46^ The NanoModeler algorithm ensured that EG chains were uniformly distributed on the AuNP surface by maximizing the distance between their sites of attachment. The validation of the implemented CG models has been fully addressed by us in ref 49, where we confirm that the CG framework adopted in this work faithfully captures the atomistic behavior for a large set of structural and energetic properties. For further information, refer to Reliability and Validation of the MARTINI CG Models, SI.

#### Linear Interpolation Model for SAM Thickness Predictions

To predict the SAM thickness corresponding to the experimental TGA grafting density of the three systems studied, namely ***homo-*EG**_**4**_, ***mix-*EG**_**4**_ **/EG**_***n***_, and ***hete-*EG**_***n***_, we implement a linear interpolation model based on the known relationships between grafting density and SAM thickness from the CG simulations. The calibration of this model was based on the reference CG grafting densities, from 2.0 to 5.0 thiol/nm^2^, and their corresponding average SAM thicknesses determined from the CG simulations. To predict the optimal SAM thicknesses that match the experimental TGA grafting densities for each one of the three experimental systems, we assumed a linear relationship between grafting density and SAM thickness within the range of simulated data, where *h*_CG_ stands for the predicted SAM thickness for a given reference experimental density σ_exp_, as seen in eq 7:

7 $$h_{CG}\left(\sigma_{\exp}\right)=h_{i}+\frac{\left(\sigma_{\exp}-\sigma_{i}\right)}{\left(\sigma_{i+1}-\sigma_{i}\right)}\left(h_{i+1}-h_{i}\right)$$

where *h*_*i*_ and *h*_*i+1*_ stand for the SAM thicknesses at the reference grafting densities *σ_i_* and *σ_i+1_*, while *h*_CG_ is the predicted thickness for a given experimental grafting density *σ_exp_*.

#### Estimation of Shear Viscosity

The shear viscosity of the refined polarizable MARTINI water was determined from EMD simulation trajectories by computing the integral of the off-diagonal components of the pressure tensor using the Einstein relation in eq 8:

$$\eta_{\alpha \beta }=\frac{1}{2}\frac{V}{\kappa_{B}T}\lim_{t\to \infty }\frac{d}{dt}\langle {\left(\int_{t_{0}}^{t_{0}+t}P_{\alpha \beta }\left(t^{\prime }\right)dt^{\prime }\right)}^{2}\rangle_{t_{0}}$$ 8

in which αβ ∈ {*xy*,*xz*,*yz*}, *V* is the system volume, κ_B_ is the Boltzmann’s constant, and *T* is the temperature. To ensure statistical robustness, ten independent simulation replicas of a cubic-shaped box of 10 × 10 × 10 nm^3^ filled with polarizable MARTINI water beads at an approximate density of 0.99 g/cm^3^ were performed. All MD simulations were carried out using the open-source GROMACS (version 2021.3) code^76^ using the refined polarizable MARTINI water force field for PME electrostatics^47^ with a time step of 20 fs. Each simulation replica began with a minimization phase using the steepest descent algorithm with a number of 50,000 steps to minimize forces and the potential energy of the system. Subsequently, an equilibration phase 50 ns long was conducted in the NPT ensemble, holding constant temperature (303 K) and pressure (1 atm) using the V-rescale thermostat (τ_B_ = 1.0 ps) and the isotropic Berendsen barostat (τ_P_ = 5.0 ps and κ = 4.5 × 10^–5^ bar^–1^),^72^ respectively. This stage allowed the simulation box volume to relax and enabled each system to reach the equilibrium density of bulk-water. Following the equilibration phase, we performed a production phase 100 ns long for each simulation replica. Nonbonded interactions were computed using a potential-shift cutoff scheme with forces smoothly shifting to zero within a real-space cutoff of 1.1 nm. Long-range electrostatics were treated using the PME method with a Fourier grid spacing of 0.15 nm and the background dielectric constant set to ϵ_r_ = 2.5. Temperature was controlled using the V-rescale thermostat set at 303.15 K with a coupling constant of 1.0 ps. Pressure coupling was disabled during the production runs to maintain a NVT ensemble. PBC conditions were applied in all directions. Energy calculations were performed at every simulation step, and the average shear viscosity was estimated by postprocessing the time-integrated off-diagonal components of the pressure tensor according to the protocol described in refs 81,82 . The fitting window was defined as the longest time interval during which the normalized standard error remained below a predefined tolerance threshold of 0.1, with a lower bound on the starting time (*t*_min_ = 10 ps) imposed to avoid artifacts from early-time nondiffusive behavior. Within this interval, a weighted linear regression was applied to the average Einstein integral as a function of time, and the slope of the fit was utilized to compute the shear viscosity. To quantify the statistical uncertainty, we utilized a bootstrap resampling in each of 20 iterations, a randomized subset corresponding to 80% of the original data set was taken, and the ensemble-averaged shear viscosity was recalculated using the same fitting protocol. The standard deviation of the viscosity values obtained from these bootstrap samples was taken as the estimate of the statistical error. The average shear viscosity and its standard deviation predicted over all 10 EMD simulation replicas for the refined polarizable MARTINI water model is 3.0 × 10^–4^ ± 1.1 Pa · s.

## Supplementary Material



## Notes

Materials and general methods, list of abbreviations, synthesis of PEGylated alkyl thiols, preparation and characterization of AuNPs, supporting computational data, cell cultures and nanoparticle internalization experiments, and NMR spectra (PDF)
