# Supplementary material for: Controlling Macrophage Uptake of Gold Nanoparticles through the Design of an Effective Heterogeneous Coating
Source: ACS Appl Mater Interfaces. 2026 Apr 28;18(18):25847–62. doi: 10.1021/acsami.6c01799 (PMC13181713; doi:10.1021/acsami.6c01799)
Supplement: Supplementary file 1 [file am6c01799_si_001.pdf]

# Controlling Macrophage Uptake of Gold Nanoparticles through the Design of an Effective Heterogeneous Coating


*Paulo Siani[a*], Ander Eguskiza [b], Giulia Frigerio[a], Edoardo Donadoni[a], Riccardo Ossanna[b], Martin Volk [c], Mathias Brust [c], Barbara Giovannone[b], Roberto Fiammengo[b*], and Cristiana Di Valentin[a*]*

[a] *Department of Materials Science, University of Milano-Bicocca, via R. Cozzi 55, 20125 Milan, Italy.*
*E-mail: paulo.siani@unimib.it; cristiana.divalentin@unimib.it*

[b] *Department of Biotechnology, University of Verona, Strada Le Grazie 15, 37134 Verona, Italy.*
*E-mail: roberto.fiammengo@univr.it*

[c] *Department of Chemistry, University of Liverpool, Liverpool L69 7ZD, UK.*


# *Supporting Information*

# *Table of contents*

**Materials and General Methods** .......... **3**

**List of Abbreviations** .......... **4**

**Synthesis of PEGylated alkyl thiols** .......... **4**

**Characterization of AuNPs** .......... **8**

*TEM* .......... *8*

*Gel electrophoresis.* .......... *8*

*Quantification of the grafting density via thermogravimetric analysis (TGA).* .......... *9*

*Quantification of mixed-SAMs composition via $^{1}H$ NMR.* .......... *10*

*Physicochemical properties of additional mixed SAM-coated AuNP formulations.* .......... *12*

*Z-potential measurements at higher ionic strength.* .......... *12*

**Supporting Computational Data** .......... **14**

*Charge Screening Profiles* .......... *14*

*Order Parameters* .......... *14*

*Computational Predictions of Zeta Potential at Varying Salt Concentrations* .......... *17*

*Radius of Gyration* .......... *18*

*Particle Count Profiles* .......... *19*

**Reliability and Validation of MARTINI CG Models** .......... **20**

**Cell Cultures and Nanoparticle Internalization experiments** .......... **21**

**References** .......... **24**

**NMR spectra** .......... **26**

## Materials and General Methods

All reagents were purchased from Sigma-Aldrich, Acros Organics, or Alfa Aesar unless mentioned otherwise, and used without further purification. Acids for ICP-MS analysis from Merck: hydrochloric acid (cat. # 1.0031, 30% Suprapur®); nitric acid (cat. # 1.15187, 69% Suprapur®); Element reference solutions for ICP-MS analysis from Romil: gold (cat. # E3AU6, 1000 ppm Au) and bismuth (cat. # E3Bi6, 1000 ppm Bi). Alkyl-$PEG_{600}$ thiol ((1-mercaptoundec-11-yl)$PEG_{600}$)-acetic acid **2** was prepared according to literature.[1]. All glassware employed for nanoparticle preparation was cleaned with aqua regia (3:1 v/v HCl (37%)/$HNO_3$ (65%)). Ultrapure deionized water (Milli-Q Advantage A10 Water Purification System, 18.2 MΩ cm) was used for the preparation of all aqueous solutions. All solutions used for nanoparticle preparation were filtered through 0.22 µm membrane filters (cellulose acetate, Whatman Puradisc 30/0.2 or Corning® cat. # 430513). AuNP core diameter was measured with a Tecnai G 2 (FEI) TEM operating at 100 kV and images were acquired with a Veleta digital camera (Olympus Soft Imaging System). UV/Vis measurements were carried out using a TECAN Infinite M200 Pro plate reader. The concentration of AuNPs was determined by measuring the absorbance at 520 nm of samples dispersed in water in comparison with AuNP samples whose concentration was independently quantified via inductively coupled plasma-optical emission spectrometry (ICP-OES) assuming the nanoparticles were spherical.[2] NMR experiments were performed at 25 °C using a 400 MHz Bruker Avance III HD spectrometer or a 600 MHz Bruker Avance III spectrometer equipped with a triple resonance TCI cryogenic probe. $^{1}H$ and $^{13}C\{^{1}H\}$ NMR spectra were calibrated to TMS based on the relative chemical shift of the solvent as an internal standard. Abbreviations used are as follows: s = singlet, d = doublet, t = triplet, q = quartet, m = multiplet. MALDI mass spectra were acquired on an ultrafleXtreme MALDI-TOF/TOF mass spectrometer (Bruker). TLC analyses were carried out

using silica gel plates Polygram® Sil G/UV254 (40×80 mm) from Macherey-Nagel. Flash chromatography was performed on silica gel 40 – 63 µm (230 – 400 mesh) from Macherey-Nagel.

## List of Abbreviations

$EG_4$ thiol= thiol **1** (see **Scheme S1**), $EG_n$ thiol= thiol mixture **2** (see **Figure 1** main text), EA=ethyl acetate, Hex=hexane, DCM=dichloromethane, THF=tetrahydrofuran, DMF=*N*,*N*-dimethylformamide, ACN=acetonitrile, EtOH=ethanol, MeOH= methanol, TFA=trifluoroacetic acid, AcOH=acetic acid.

## Synthesis of PEGylated alkyl thiols

**Scheme S1.** Synthesis of ((1-mercaptoundec-11-yl)tetra(ethylene glycol))-3-propionic acid (**1**).[a]

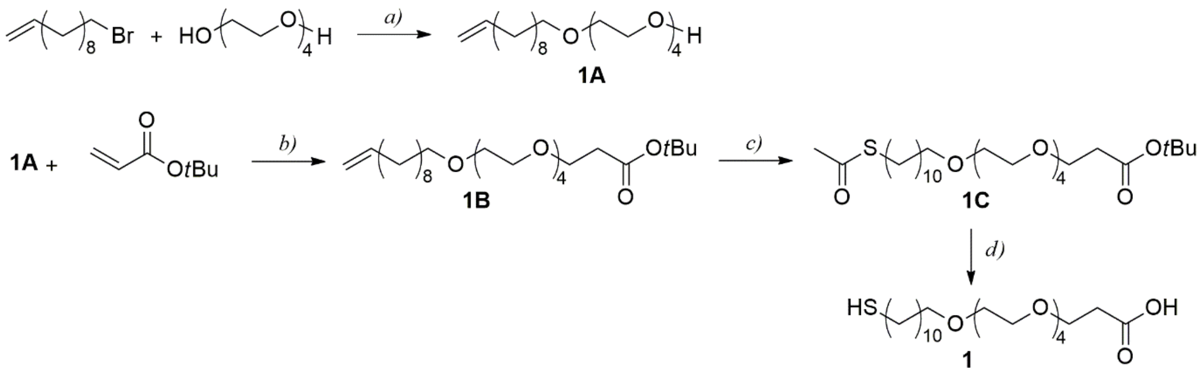


[a]Reagents and conditions: (*a*) tetraethylene glycol (4.0 equiv.), NaH (1.2 equiv.), THF, 60 °C, 3 h, 70%; (*b*) *t*-Butylacrylate (1.4 equiv.), *t*BuOK (0.03 equiv.), THF, r.t., overnight, 63%; (*c*) thioacetic acid (4.0 equiv.), BAPO (0.05 equiv.), MeOH, r.t., *hν* (395 nm 6.0 W/cm²), 1 h, 92%; (*d*) 3 M NaOH/MeOH 1:1, r.t., 3 h, 80%. BAPO=*bis*(2,4,6-trimethylbenzoyl)phenylphosphine oxide.

***Undec-1-en-11-yl-tetraethylene glycol* (1A)**.[3] Tetraethylene glycol (11.46 g, 59.00 mmol, 4.0 equiv) was dried by co-evaporation with toluene (4×100 mL) and dissolved under argon in 90 mL of dry THF. NaH (0.705 g, 60% in oil, 17.6 mmol, 1.2 equiv) was then added and the reaction mixture was brought to 60 °C and stirred until $H_2$ evolution ceased (~20 min). Successively, a solution of 11-bromo-1-undecene (3.44 g, 14.7 mmol) in 50 mL dry THF was slowly added over 3 h using a syringe pump while keeping the reaction mixture at 60 °C. After this period, the reaction was quenched by addition of 20 mL $H_2O$ and partitioned between 200 mL EA and 200 mL $H_2O$. The aqueous phase was extracted with 100 mL EA and the combined organic layers were washed with $H_2O$ (2×200 mL) and brine (2×200 mL). Drying over $Na_2SO_4$ and evaporation of the solvent under reduced pressure afforded the crude product as a yellow oil. Purification by flash chromatography (elution: from Hex/EA to EA) gave **1A** as a pale-yellow oil (3.57 g, 10.3 mmol, 70% yield).

$^1$H NMR (400 MHz, $CDCl_3$) d 5.80 (m, 1 H), 5.02-4.89 (m, 2H), 3.72 (m, 2H), 3.69-3.55 (m, 14H), 3.44 (t, *J* = 6.8 Hz, 2H), 2.66 (s, 1H), 2.03 (m, 2H), 1.57 (m, 2H), 1.41-1.22 (m, 12H).

$^{13}$C NMR (100 MHz, $CDCl_3$) d 139.14, 114.02, 72.53, 71.48, 70.54, 70.52, 70.49, 70.46, 70.22, 69.96, 61.63, 33.72, 29.49, 29.45, 29.37, 29.35, 29.04, 28.84, 25.98.

***((Undec-1-en-11-yl)tetraethylene glycol)-3-propionic acid t-butyl ester* (1B)**. Undec-1-en-11-yl-tetraethylene glycol **1A** (2.11 g, 6.08 mmol) was dried by co-evaporation with toluene (4×50 mL), dissolved under argon in 50 mL of dry THF, and potassium *t*-butoxide (22.2 mg in 2 mL dry THF, 0.198 mmol, 0.03 equiv.) was added. After 10 min. stirring, *t*-butylacrylate (1.2 mL, 8.27 mmol, 1.4 equiv.) was added and the reaction mixture further stirred, protected for light, overnight at room temperature. After this time, the reaction was quenched by the addition of 2 mL of saturated

aq. $NH_4Cl$ and then partitioned between 100 mL EA and 70 mL $H_2O$. The organic phase was separated and washed with brine (2×100 mL). Drying over $Na_2SO_4$ and removal of the solvent under reduced pressure afforded the crude product as viscous pale-yellow oil. Purification by flash chromatography (elution: from Hex/EA 99:1 to Hex/EA 50:50) gave **1B** as a colorless oil (1.81 g, 3.81 mmol, 63% yield).

$^1$H NMR (400 MHz, $CDCl_3$) d 5.80 (m, 1 H), 5.02-4.89 (m, 2H), 3.70 (t, $J$ = 6.6 Hz, 2H), 3.66-3.55 (m, 16H), 3.44 (t, $J$ = 6.8 Hz, 2H), 2.50 (t, $J$ = 6.6 Hz, 2H), 2.03 (m, 2H), 1.57 (m, 2H), 1.44 (s, 9H), 1.40-1.24 (m, 12H).

$^{13}$C NMR (100 MHz, $CDCl_3$) d 170.81, 139.12, 114.03, 80.39, 71.45, 70.55, 70.52, 70.43, 70.29, 69.98, 66.82, 36.19, 33.72, 29.55, 29.45, 29.38, 29.34, 29.03, 28.84, 28.02, 26.00.

***([1-[(Methylcarbonyl)thio]undec-11-yl]tetraethylene glycol)-3-propionic acid t-butyl ester*** **(1C)**. ((Undec-1-en-11-yl)tetraethylene glycol)-3-propionic acid t-butyl ester **1B** (1.67 g, 3.52 mmol) was dissolved in 25 mL of methanol. The solution was degassed by 4 freeze-pump-thaw cycles and placed under argon. *Bis*(2,4,6-trimethylbenzoyl)phenylphosphine oxide (BAPO, 67 mg, 0.16 mmol, 0.05 equiv) and thioacetic acid (1.0 mL, 13.9 mmol, 4.0 equiv) were added. The reaction mixture was irradiated at 395 nm using a UV LED lamp (IRIS 70, peak radiance 6.0 W/cm$^2$, Photo Electronics Srl, Italy) for 1 h at room temperature. After this time, the volatiles were removed under reduced pressure and the residue purified by flash chromatography (elution: from Hex/EA 98:2 to Hex/EA 50:50) affording product **1C** as a colorless oil (1.79 g, 3.25 mmol, 92% yield).

$^1$H NMR (400 MHz, $CDCl_3$) d 3.70 (t, $J$ = 6.6 Hz, 2H), 3.67-3.55 (m, 16H), 3.44 (t, $J$ = 6.8 Hz, 2H), 2.85 (t, $J$ = 7.4 Hz, 2H), 2.49 (t, $J$ = 6.6 Hz, 2H), 2.31 (s, 3H), 1.60-1.51 (m, 4H), 1.44 (s, 9H), 1.36-1.22 (m, 14H).

$^{13}C$ NMR (100 MHz, $CDCl_3$) d 195.88, 170.78, 80.36, 71.42, 70.51, 70.49, 70.40, 70.27, 69.95, 66.79, 36.16, 30.53, 29.52, 29.44, 29.39, 29.35, 29.34, 29.03, 28.89, 28.70, 27.99, 25.97.

***((1-mercaptoundec-11-yl)tetra(ethylene glycol))-3-propionic acid*** **(1)**. The protected thiol precursor **1C** (1.66 g, 3.01 mmol) was dissolved in 16 mL of methanol in a Schlenk flask under argon atmosphere and 16 mL of a 3 M NaOH solution were quickly added. The mixture was immediately degassed by 4 freeze-pump-thaw cycles and placed under argon. After 3 h stirring at room temperature, the reaction mixture was acidified by addition of 4.5 mL of 36% conc. HCl, poured into 150 mL brine and extracted with 150 mL EA. The organic layer was washed with 150 mL brine, dried over $Na_2SO_4$ and the solvent removed under vacuum. The crude product was purified by flash chromatography (elution: from Hex/EA/AcOH 50:50:1 to Hex/EA/AcOH 70:30:1) to give **1** as white waxy solid (1.12 g, 2.47 mmol, 82% yield).

$^{1}H$ NMR (400 MHz, $CDCl_3$) d 3.77 (t, $J$ = 6.1 Hz, 2H), 3.69-3.57 (m, 16H), 3.44 (t, $J$ = 6.8 Hz, 2H), 2.61 (t, $J$ = 6.1 Hz, 2H), 2.51 (q, $J$ = 7.4 Hz, 2H), 1.64-1.51 (m, 4H), 1.41-1.21 (m, 15H).

MALDI-TOF-MS ($C_{22}H_{44}O_7SNa$) *m/z* 474.9929 ($[M+Na]^+$, calc. 475.2700).

## Characterization of AuNPs

*TEM*

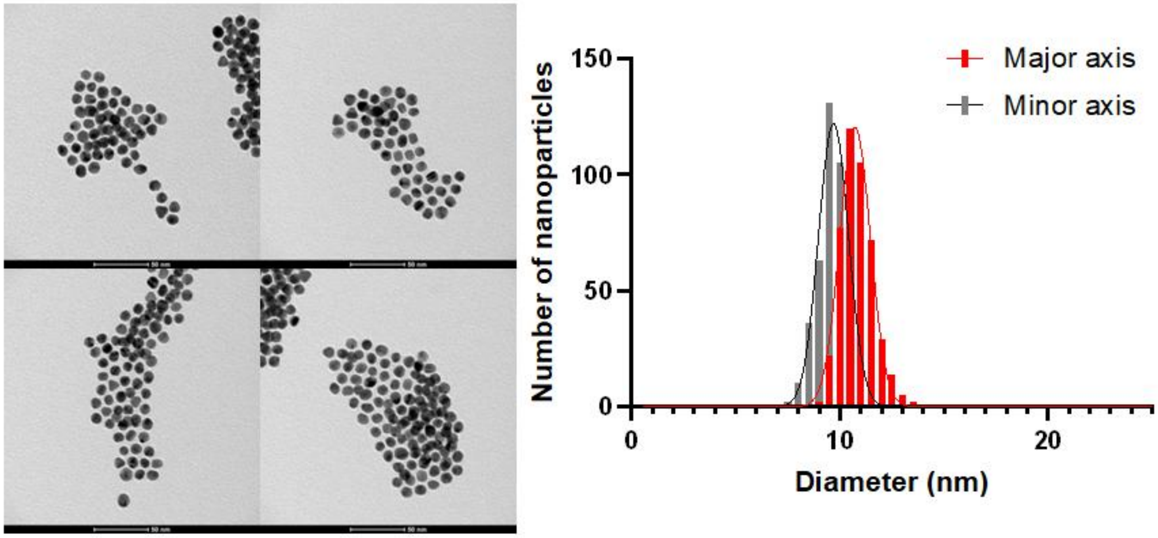


**Figure S1.** Left: Representative TEM micrographs of the gold nanoparticles (AuNPs) employed in this study coated with polydisperse $EG_n$ thiol **2**. Right: results of the TEM image analysis on 450 nanoparticles: AuNPs were fitted to ellipses and image analysis afforded for each AuNP *major* and *minor* axis of the ellipse, whose distributions are plotted in the graph. The *ellipticity* ($= \frac{major\ axis}{minor\ axis}$) of the synthesized AuNPs is ~1.1, which justify the assumption of spherical nanoparticles. The diameter reported in the text (10.2 ± 0.7 nm) is the average of the *major* and *minor* axis determined from the shown distributions given the very similar s.d. for the two distributions.

*Gel electrophoresis.*

Gel electrophoresis was carried out on 0.6% agarose gel using 5 mM sodium boric acid (SB) buffer pH 8.5, for approx. 2 h at 60 V.[4] AuNPs coated with (mixed) SAMs of **$EG_4$** thiol (**1**) and polydisperse **$EG_n$** thiol (**2**) with a molar fraction of **1** ($\chi_1$) as indicated on **Figure 2D** (main text) were diluted with loading buffer (1:1 SB buffer/glycerol) to 25 nM AuNPs before loading on the

gel (~10 μL/lane). Only very minor differences in electrophoretic mobility are observed for the 5 different formulations loaded on the gel.

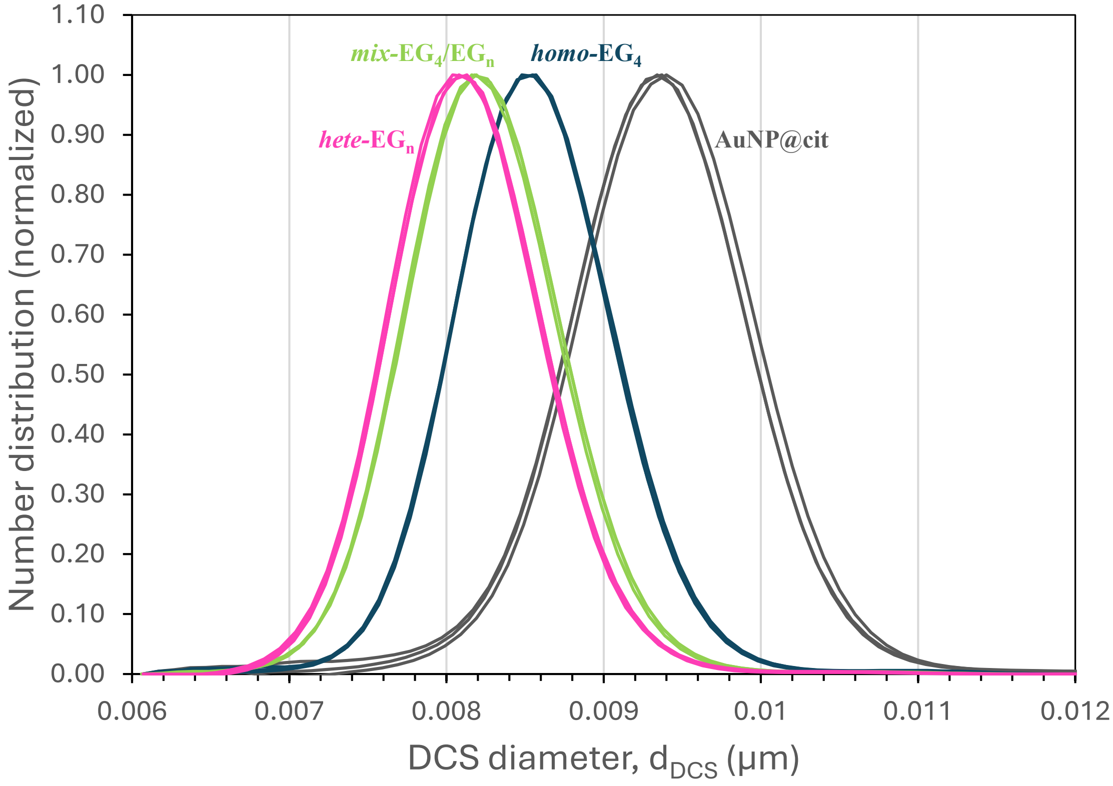


**Figure S2.** Normalized number size distribution for **AuNP@cit**, ***homo*-EG4, *mix*-EG4/ EGn**, ***hete*-EGn** analyzed by DCS. For each AuNP formulation three runs were performed and are shown with lines of the same color in this figure. The reported coating thicknesses are calculated according to literature[5,6] assuming a density of 1.12 g/cm$^3$ for both EG4 thiol **1** and EGn thiol mixture **2**.[7]

## *Quantification of the grafting density via thermogravimetric analysis (TGA).*

Measurements were carried out using a Q5000IR TGA instrument (TA Waters) or a TGA-DSC 3+ instrument (Mettler Toledo). An aliquot of each sample (ca. 150 to 200 μL of AuNPs solution at a concentration 250 – 350 nM in milliQ water) was placed in an alumina crucible, dried to constant weight at 105 °C, and then heated to 700 °C at 10 °C/min under an atmosphere of synthetic air at a flow rate of 25 mL/min. The grafting density was obtained dividing the number of thiol

molecules in a sample by the total nanoparticle surface area. Briefly, the total mass of organic matter in the sample was calculated as a difference from the dry mass of the sample at 105 °C and the constant mass remaining at the end of the analysis (only gold). The dry mass of the samples was 0.735 – 1.040 mg; the lowest amount of organic matter was around 9% of the dry mass and the instrumental weighing precision around 10 μg.

The mass obtained was attributed to the coating layers, whose molecular identity (the thiol molecules) is known. The number of nanoparticles in a sample was calculated from the measured gold mass remaining in the crucible by dividing for the mass of a single nanoparticle modelled as a sphere with diameter determined via TEM analysis. From the number of nanoparticles in a sample, the total nanoparticle surface area and the grafting density were finally calculated. The results for multiple batches of nanoparticles independently prepared are shown in **Figure 2E** (main text). Statistical analysis of the results was performed via ordinary one-way ANOVA and post-hoc Tukey's tests and showed no significant differences in grafting density.

## *Quantification of mixed-SAMs composition via $^1$H NMR.*

Sample preparation: 200 μL of coated AuNPs dispersed in milliQ water at a concentration of ~200 nM were mixed with 200 μL of $CD_3OD$ in glass vials. Approximately 200 μL of a 32 mM $I_2$ solution in $CD_3OD$ were added to each sample to etch the gold core. Samples were thoroughly mixed and placed closed in an ultrasonic bath at r.t. for a total of 8 minutes followed by brief centrifugation. 500 μL of each solution were placed in a 5 mm NMR tube for measurement.

Acquisition of $^1$H NMR spectra: spectra were acquired using a Bruker Ascend 600 MHz spectrometer equipped with a TCI cryoprobe at 298K using the standard Bruker pulse sequence "zgesp" for water suppression and using a 9615.38 Hz sweep width, 32K data points, 32 scans and

a relaxation delay of 10s. Quantification of the amount of $EG_4$ thiol **1** and $EG_n$ thiols **2** released from the etched gold core (all released as disulfides) was done integrating the signal for the -C**H**$_2$- next to the sulfur atom (overlapped for **1** and **2**) *vs.* the signal for the $OCH_2$-C**H**$_2$-COOH (only for **1**) as shown in **Figure S3**. According to this quantification, the ***mix*-EG$_4$/EG$_n$** SAM contained 52% of $EG_4$ thiol **1** and 48% of $EG_n$ thiols **2** matching well the molar fraction of the two components used for during the passivation reaction ($\chi_1 = 0.50$, **Figure 1** main text).

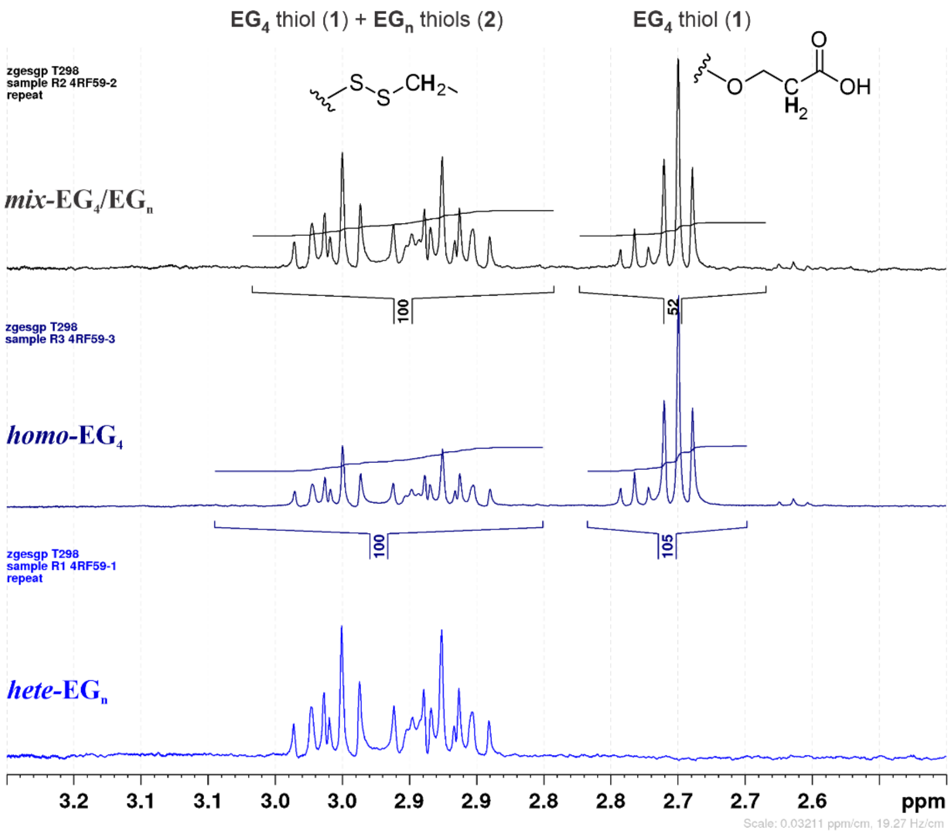


**Figure S3.** Quantification of the amount of $EG_4$ thiol **1** and $EG_n$ thiols **2** released as disulfides from the etched gold core of ***mix*-EG$_4$/EG$_n$**, ***homo*-EG$_4$**, and ***hete*-EG$_n$** AuNPs via $^1$H NMR in $H_2O/CD_3OD$ at 600 MHz, r.t.

## *Physicochemical properties of additional mixed SAM-coated AuNP formulations.*

**Table S1.** Physicochemical characteristics of AuNPs coated by SAMs determined by dynamic light scattering (DLS) and electrophoretic light scattering (ELS).

| | $\chi_1$ | Hydrodynamic diameter (nm) | Polydispersity index (PDI) | Zeta potential (mV) |
|---|---|---|---|---|
| ***mix*-25EG$_4$/EGn** | 0.25 | 20.4 ± 0.4 | 0.278 | -45 ± 5 |
| ***mix*-75EG$_4$/EGn** | 0.75 | 20.3 ± 0.3 | 0.284 | -49 ± 1 |

## *Z-potential measurements at higher ionic strength.*

The measurement of ZP via ELS in high ionic strength solution is experimentally challenging for several reasons including non-negligible Joule heating of the solution which affects pH and viscosity of the solution,[8,9] irreversible sample degradation, and electrode blackening (degradation).[10] We measured ZPs of ***homo*-EG$_4$**, ***mix*-EG$_4$/EG$_n$**, and ***hete*-EG$_n$** AuNP in 10 mM $NaHCO_3$ +100 mM NaCl and compared with the ZPs for the same formulations in 10 mM $NaHCO_3$ using an applied voltage of 75 V. We observed a slight decrease in the measured absolute values of ZPs (Table S2), in agreement with the knowledge that increasing ionic strength results in a more compressed electric double layer and reduced absolute ZP values.

Nevertheless, these measurements should be taken with caution since significant electrode blackening (degradation) was observed upon measurement in 10 mM $NaHCO_3$ +100 mM NaCl (**Figure S4**), even if the measurements were carried out using the diffusion barrier method.[8]

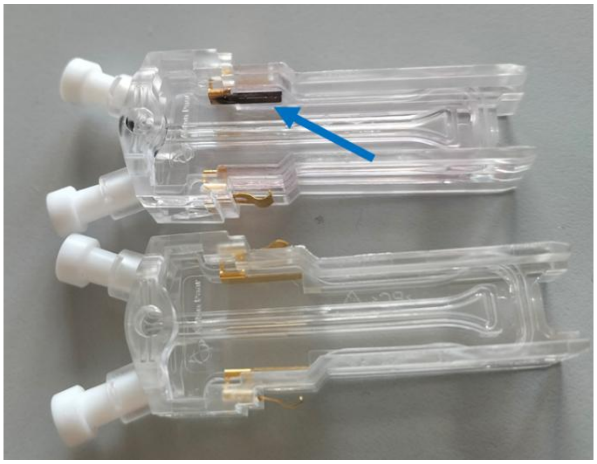

**Figure S4.** Image of two Omega Cuvettes from Anton Paar (cat. #225288) used for Z-potential measurements of SAMs coated AuNPs. The top cuvette shows a blackened electrode (blue arrow) observed after several measurements using nanoparticle solutions at higher ionic strength (10 mM $NaHCO_3$ + 100 mM NaCl, conductivity ~9.5 mS/cm) with a 75 V applied voltage. For comparison purposes, the lower cuvette has been used at the same applied voltage with nanoparticle dispersed in 10 mM $NaHCO_3$ (conductivity ~0.9 mS/cm).

**Table S2.** Zeta potential and solution conductivity for single batches of AuNPs coated by SAMs measured by electrophoretic light scattering (ELS).[a]

| Zeta potential (mV)<br>(Conductivity mS/cm) | ***homo*-EG$_4$** | ***mix*-EG$_4$/EG$_n$** | ***hete*-EG$_n$** |
|---|---|---|---|
| 10 mM $NaHCO_3$ | -41 ± 6<br>(0.836) | -37 ± 8<br>(0.873) | -37 ± 7<br>(0.838) |
| 10 mM $NaHCO_3$<br>+100 mM NaCl | -38 ± 6<br>(9.46) | -34 ± 7<br>(9.99) | -32 ± 7<br>(9.55) |

## Supporting Computational Data

### *Charge Screening Profiles*

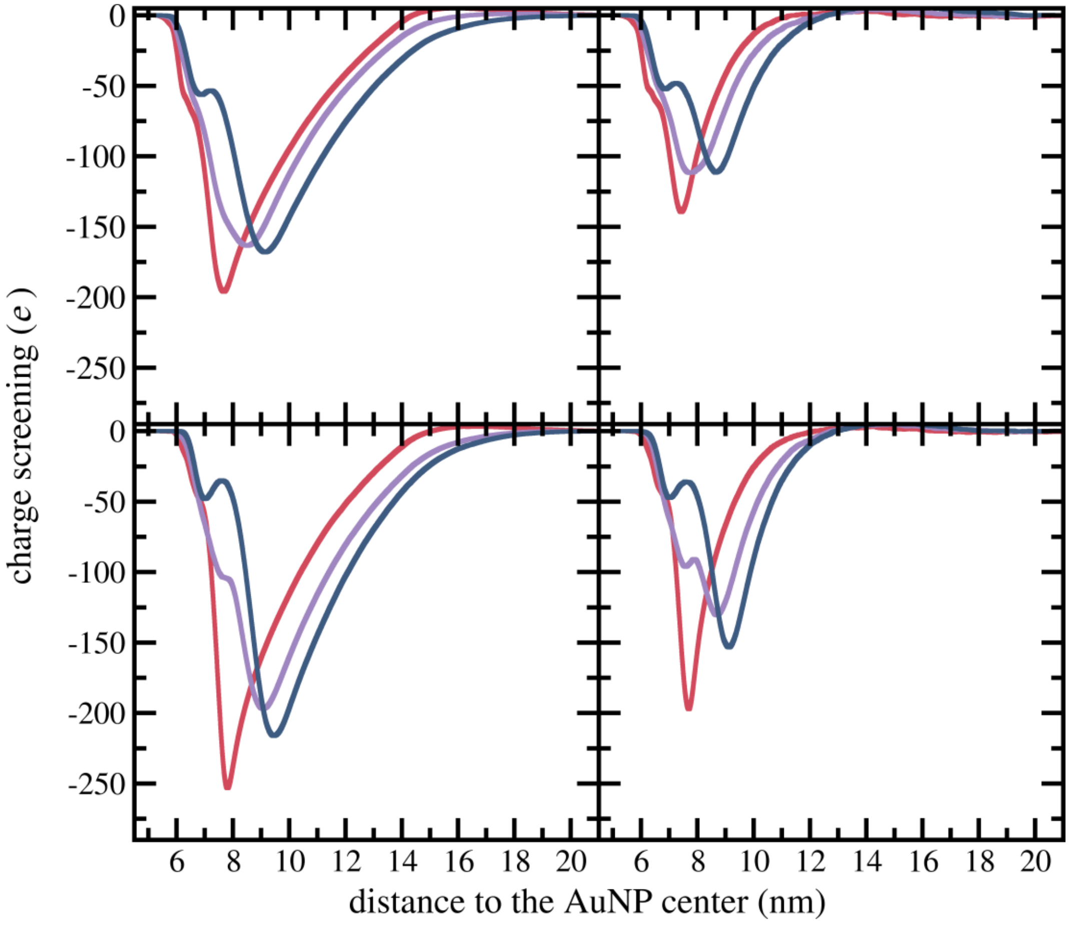


**Figure S5.** Radial charge screening profiles of ***homo*-$EG_4$** (red), ***mix*-$EG_4$/$EG_n$** (violet), and ***hete*-$EG_n$** (blue) AuNPs in 10 mM (left-hand side panels) and 150 mM (right-hand side panels) NaCl solution. Top panels: grafting density at 2.0 thiols/$nm^2$; Bottom panels: 3.0 thiols/$nm^2$.

### *Order Parameters*

**Tables S3 and S4** present the ordering parameter values, $\langle S \rangle$, for $EG_4$ and $EG_n$ chains making up the coating of ***homo*-$EG_4$**, ***mix*-$EG_4$/$EG_n$**, and ***hete*-$EG_n$**. $\langle S \rangle = 1$ means perfect alignment, and therefore, an anisotropic orientational behavior of the $\vec{n}$ vector to the principal axis vector, $\langle S \rangle =$

0 indicates random alignment, and therefore, an isotropic orientational behavior of the $\vec{n}$ vector to the principal axis, and negative $\langle S \rangle$ values indicate a preference for a perpendicular alignment of $\vec{n}$ vectors to the principal axis vector.

**Table S3.** Average $\langle S \rangle$ values for all $\vec{n}$ vectors connecting identical CG bead pairs of $EG_4$ chains within the ***homo*-EG₄** coating. Averages are taken across all $EG_4$ chains with matching bead-bead $\vec{n}$ vectors.

| bead-bead $\vec{n}$ vector | *homo*-$EG_4$ | | | | *mix*-$EG_4/EG_n$ | | | |
|---|---|---|---|---|---|---|---|---|
| | *2 thiols/nm²* | *3 thiols/nm²* | *4 thiols/nm²* | *5 thiols/nm²* | *2 thiols/nm²* | *3 thiols/nm²* | *4 thiols/nm²* | *5 thiols/nm²* |
| $C1_1$-$C1_2$ | 0.005±0.009 | 0.250±0.009 | 0.491±0.001 | 0.750±0.001 | 0.053±0.029 | 0.294±0.011 | 0.484±0.002 | 0.734±0.001 |
| $C1_2$-$C1_3$ | -0.118±0.011 | 0.148±0.011 | 0.398±0.001 | 0.645±0.001 | -0.001±0.015 | 0.231±0.009 | 0.378±0.003 | 0.605±0.001 |
| $C1_3$-$EO_1$ | -0.093±0.013 | 0.159±0.011 | 0.385±0.001 | 0.585±0.001 | 0.031±0.016 | 0.227±0.005 | 0.349±0.004 | 0.524±0.002 |
| $EO_1$-$EO_2$ | -0.053±0.015 | 0.053±0.013 | 0.152±0.001 | 0.246±0.001 | 0.005±0.007 | 0.066±0.003 | 0.097±0.003 | 0.167±0.002 |
| $EO_2$-$EO_3$ | 0.011±0.016 | 0.161±0.014 | 0.277±0.001 | 0.372±0.001 | 0.097±0.012 | 0.182±0.003 | 0.224±0.001 | 0.304±0.002 |
| $EO_3$-$EO_4$ | 0.170±0.017 | 0.286±0.014 | 0.361±0.001 | 0.411±0.001 | 0.205±0.008 | 0.276±0.002 | 0.332±0.002 | 0.386±0.001 |
| $EO_4$-$EO_5$ | 0.256±0.019 | 0.297±0.015 | 0.361±0.001 | 0.428±0.001 | 0.208±0.004 | 0.248±0.003 | 0.319±0.001 | 0.383±0.001 |
| $EO_5$-Qa | 0.237±0.018 | 0.259±0.015 | 0.316±0.001 | 0.389±0.001 | 0.186±0.005 | 0.216±0.002 | 0.283±0.001 | 0.348±0.001 |

**Table S4.** Average $\langle S \rangle$ values for all $\vec{n}$ vectors connecting identical CG bead pairs of $EG_4$ and $EG_n$ chains within the ***mix*-EG4/EGn**, and ***hete*-EGn** coatings. Averages are taken across all $EG_4$ and $EG_n$ chains with matching bead-bead $\vec{n}$ vectors, regardless of $EG_n$ chain length.

| **Bead-Bead $\vec{n}$ vector** | ***mix*-$EG_4/EG_n$** | | | | ***hete*-$EG_n$** | | | |
|---|---|---|---|---|---|---|---|---|
| | ***2 thiols/nm²*** | ***3 thiols/nm²*** | ***4 thiols/nm²*** | ***5 thiols/nm²*** | ***2 thiols/nm²*** | ***3 thiols/nm²*** | ***4 thiols/nm²*** | ***5 thiols/nm²*** |
| $C1_1$-$C1_2$ | 0.061±0.025 | 0.306±0.013 | 0.512±0.007 | 0.745±0.005 | 0.167±0.013 | 0.373±0.010 | 0.564±0.007 | 0.754±0.005 |
| $C1_2$-$C1_3$ | -0.021±0.014 | 0.235±0.013 | 0.422±0.009 | 0.634±0.007 | 0.034±0.015 | 0.308±0.012 | 0.509±0.010 | 0.673±0.008 |
| $C1_3$-$EO_1$ | -0.000±0.016 | 0.232±0.013 | 0.400±0.010 | 0.570±0.007 | 0.041±0.016 | 0.296±0.012 | 0.486±0.009 | 0.629±0.008 |
| $EO_1$-$EO_2$ | -0.021±0.016 | 0.080±0.014 | 0.152±0.012 | 0.234±0.010 | -0.001±0.016 | 0.117±0.014 | 0.212±0.012 | 0.300±0.010 |
| $EO_2$-$EO_3$ | 0.058±0.017 | 0.186±0.014 | 0.280±0.012 | 0.380±0.011 | 0.053±0.016 | 0.193±0.013 | 0.302±0.012 | 0.395±0.011 |
| $EO_3$-$EO_4$ | 0.131±0.018 | 0.238±0.015 | 0.327±0.013 | 0.395±0.010 | 0.073±0.018 | 0.181±0.014 | 0.265±0.013 | 0.323±0.010 |
| $EO_4$-$EO_5$ | 0.128±0.019 | 0.217±0.015 | 0.315±0.013 | 0.393±0.010 | 0.068±0.018 | 0.161±0.015 | 0.249±0.013 | 0.310±0.011 |
| $EO_5$-$EO_6$ | 0.113±0.019 | 0.194±0.015 | 0.288±0.013 | 0.371±0.011 | 0.067±0.018 | 0.153±0.015 | 0.239±0.013 | 0.305±0.011 |
| $EO_6$-$EO_7$ | 0.041±0.026 | 0.156±0.021 | 0.258±0.018 | 0.344±0.015 | 0.067±0.018 | 0.150±0.015 | 0.229±0.013 | 0.297±0.011 |
| $EO_7$-$EO_8$ | 0.056±0.026 | 0.141±0.022 | 0.219±0.018 | 0.279±0.016 | 0.072±0.018 | 0.151±0.015 | 0.224±0.013 | 0.290±0.011 |
| $EO_8$-$EO_9$ | 0.075±0.025 | 0.132±0.022 | 0.188±0.019 | 0.226±0.016 | 0.084±0.018 | 0.149±0.015 | 0.217±0.013 | 0.278±0.011 |
| $EO_9$-$EO_{10}$ | 0.091±0.026 | 0.128±0.022 | 0.164±0.019 | 0.194±0.016 | 0.095±0.018 | 0.141±0.015 | 0.201±0.013 | 0.260±0.011 |
| $EO_{10}$-$EO_{11}$ | 0.103±0.030 | 0.124±0.022 | 0.143±0.019 | 0.169±0.017 | 0.100±0.019 | 0.133±0.016 | 0.185±0.014 | 0.243±0.012 |
| $EO_{11}$-$EO_{12}$ | 0.108±0.030 | 0.116±0.024 | 0.125±0.021 | 0.144±0.019 | 0.097±0.020 | 0.123±0.016 | 0.168±0.015 | 0.224±0.013 |
| $EO_{12}$-$EO_{13}$ | 0.116±0.035 | 0.102±0.028 | 0.102±0.025 | 0.119±0.022 | 0.090±0.025 | 0.108±0.020 | 0.143±0.018 | 0.197±0.015 |
| $EO_{13}$-$EO_{14}$ | 0.114±0.043 | 0.089±0.034 | 0.083±0.031 | 0.098±0.028 | 0.080±0.030 | 0.091±0.025 | 0.119±0.021 | 0.168±0.020 |
| $EO_{14}$-$EO_{15}$ | 0.118±0.066 | 0.077±0.057 | 0.076±0.048 | 0.090±0.044 | 0.080±0.048 | 0.077±0.041 | 0.106±0.036 | 0.151±0.031 |
| $EO_{15}$-Qa | 0.099±0.207 | 0.071±0.163 | 0.063±0.154 | 0.082±0.135 | 0.123±0.144 | 0.073±0.116 | 0.096±0.109 | 0.136±0.098 |

*Computational Predictions of Zeta Potential at Varying Salt Concentrations*

**Tables S5** and **S6** present the predicted zeta potential values for 10 mM and 150 mM NaCl concentrations, calculated using the Helmholtz–Smoluchowski equation. The input parameters for these calculations include the electrophoretic mobility of *homo*-$EG_4$, *mix*-$EG_4$/$EG_n$, and *hete*-$EG_n$ AuNPs obtained from *in silico* electrophoretic Non-Equilibrium Molecular Dynamics (NEMD) simulations, as well as the relative dielectric constant and shear viscosity estimated for the refined polarizable MARTINI water model. Full details of these calculations are provided in the *Computational Methods* section.

**Table S5.** Zeta potential of AuNPs coated by SAMs predicted by non-equilibrium electrophoretic simulations at 10 mM of NaCl.

| Zeta potential (mV) | ***homo*-EG$_4$** | ***mix*-EG$_4$/EG$_n$** | ***hete*-EG$_n$** |
|---|---|---|---|
| 2 thiols/nm$^2$ | -40.2 ± 2.7 | -37.4 ± 2.5 | -35.4 ± 2.3 |
| 3 thiols/nm$^2$ | -38.9 ± 2.6 | -35.8 ± 2.3 | -34.9 ± 2.2 |
| 4 thiols/nm$^2$ | -37.4 ± 1.7 | -34.9 ± 1.6 | -33.7 ± 1.6 |
| 5 thiols/nm$^2$ | -35.9 ± 1.7 | -33.8 ± 1.5 | -32.6 ± 1.4 |

**Table S6.** Zeta potential of AuNPs coated by SAMs predicted by non-equilibrium electrophoretic simulations at 150 mM of NaCl.

| Zeta potential (mV) | ***homo*-EG$_4$** | ***mix*-EG$_4$/EG$_n$** | ***hete*-EG$_n$** |
|---|---|---|---|
| 2 thiols/nm$^2$ | -28.3 ± 2.4 | -25.5 ± 2.2 | -23.2 ± 2.1 |
| 3 thiols/nm$^2$ | -27.5 ± 2.3 | -24.5 ± 2.2 | -23.9 ± 2.0 |

*Radius of Gyration*

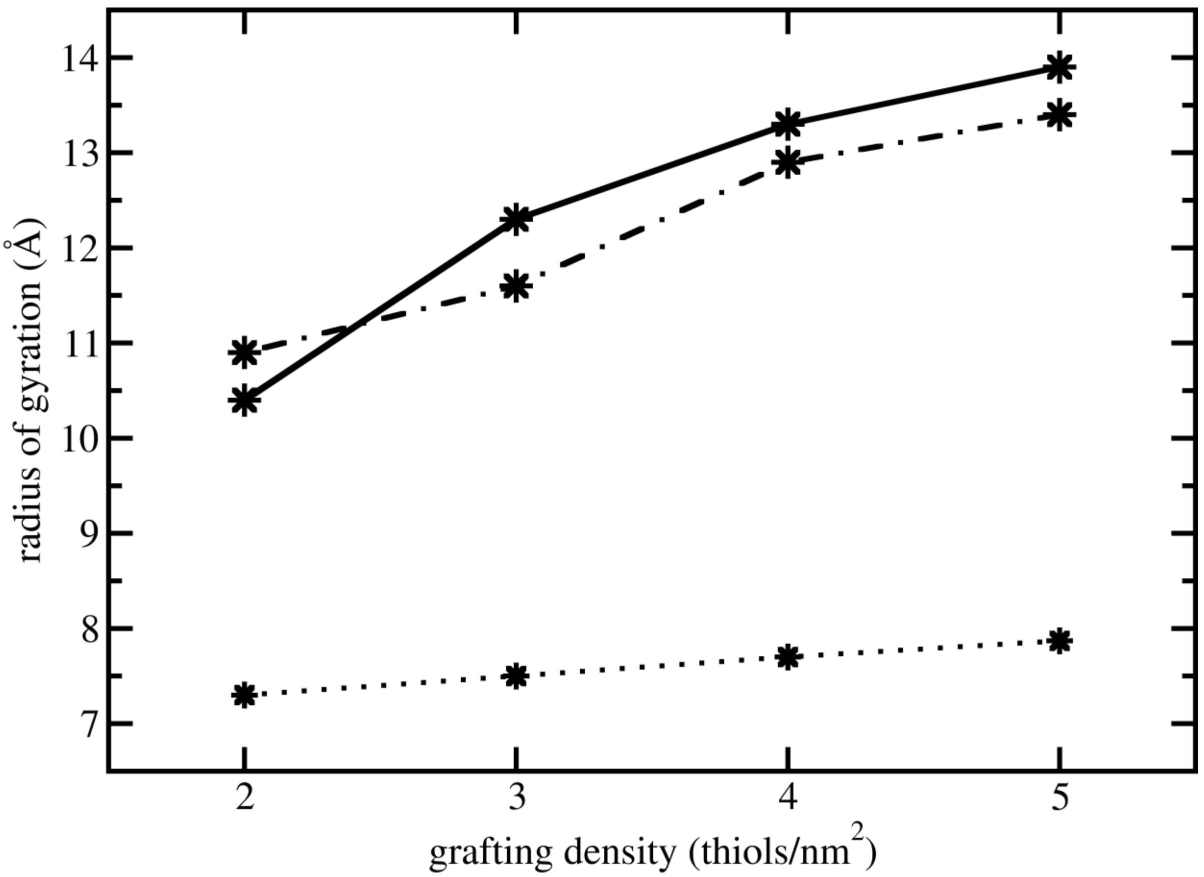


**Figure S6.** Radius of gyration (RoG) of $EG_4$ in *homo*-$EG_4$ (dotted line) and $EG_{17}$ chains in *mix*-$EG_4$/$EG_n$ (dot-dashed line) and *hete*-$EG_n$ (solid line) coatings at grafting densities of 2.0, 3.0, 4.0 and 5.0 thiols/nm$^2$.

*Particle Count Profiles*

Calculated radial charge screening profiles for ***homo*-EG4**, ***mix*-EG4/EGn**, and ***hete*-EGn** coated AuNPs in 10 mM NaCl (see Eq. 6 main text). The graphs show the number of charged beads (namely $COO^-$ groups and $Cl^-$ ions for the negative charges and $Na^+$ ions) moving from the nanoparticle center towards the bulk solvent.

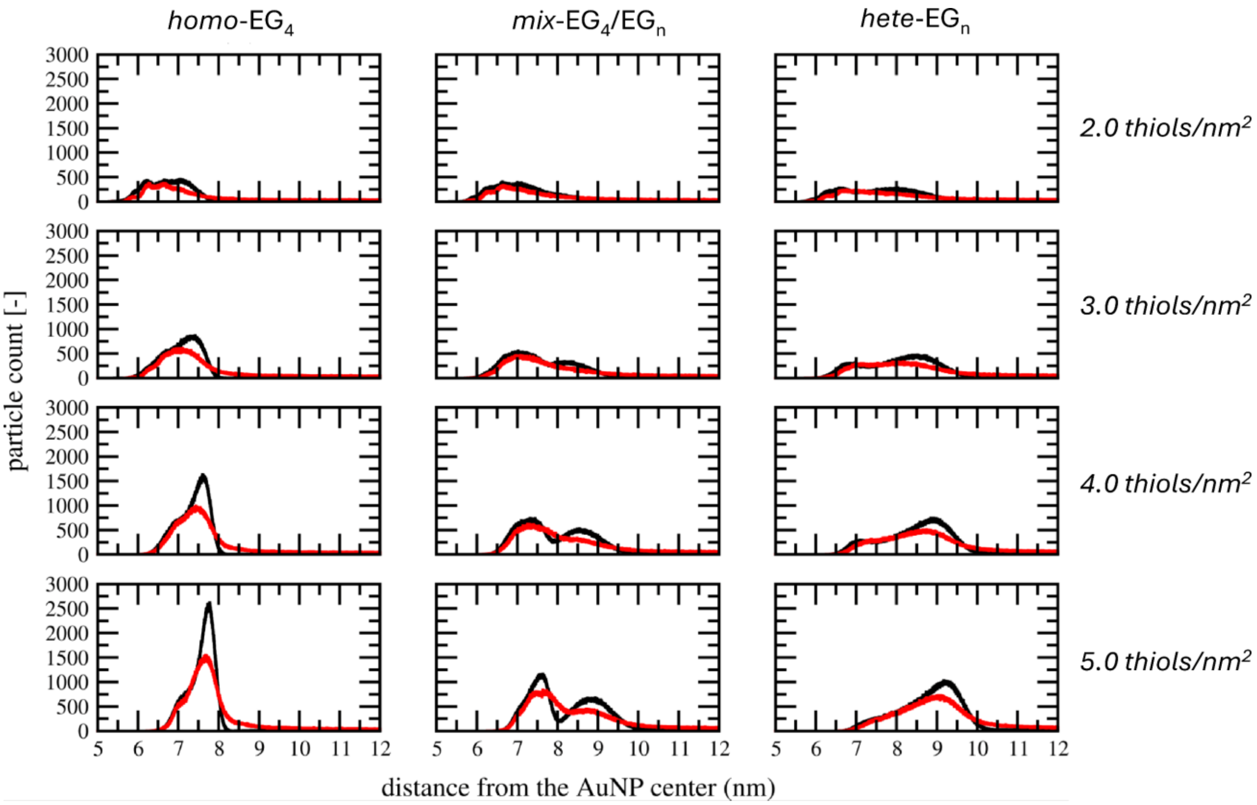


**Figure S7.** Particle count profiles of negatively (black) and positively (red) charged particles for the AuNP systems: ***homo*-EG4**, ***mix*-EG4/EGn** , and ***hete*-EGn** as indicated by column labels, shown for grafting densities of 2.0, 3.0, 4.0 thiols/nm$^2$, and 5.0 thiols/nm$^2$.

## Reliability and Validation of MARTINI CG Models

The reliability of the CG model employed in this study was previously validated against its all-atom (AA) counterpart in a dedicated publication by us,[11] using the same ligand chemistry as the *homo*-$EG_4$ systems investigated here. In that work, a smaller Au core was adopted in the AA simulations due to the computational expense of reaching microsecond time scales at atomistic resolution, yet the CG protocol accurately reproduced from the corresponding AA structural descriptors. Notably, the CG SAM thickness differed from the AA value by only 0.15 nm at the high grafting density regime of 5 thiols/nm², and polymer stretching showed close agreement, with RoG values of $0.73 \pm 0.03$ nm (CG) and $0.67 \pm 0.06$ nm (AA). Consistent log–log number-density profiles were also observed across the different grafting density regime (1 and 5 thiols/nm²). These results demonstrated that the smoother potentials and reduced anisotropy intrinsic to CG models do not disrupt the underlying structural trends, thereby confirming that the CG framework faithfully captures AA behavior for polymer stretching, terminal-group distributions, conformational regimes, solvation energies, RoG, and SAM thickness. This prior validation supports the use of the CG model for the different grafting density regimes examined in the present work. Finally, our choice of the MARTINI 2.0 force field was primarily motivated by its compatibility with the polarizable MARTINI water model, which enables a more realistic treatment of long-range electrostatics, reproducing well the dielectric response of water more faithfully than the non-polarizable variant. This is critical for capturing macroscopic solution properties such as dielectric constant and therefore in describing the electrophoretic mobility of SAM-coated Au nanoparticles in NEMD simulations.

## Cell Cultures and Nanoparticle Internalization experiments

Cells of the murine macrophage cell line RAW264.7 (TIB-71™ from ATCC) were grown in complete medium (Dulbecco's Modified Eagle's Medium – DMEM +10% Fetal Bovine Serum – FBS) at 37 °C under a humidified controlled atmosphere with 5% $CO_2$ until sub-confluence and propagated according to the specific ATCC guidelines for this product.

For the internalization experiments, cells were seeded into 6-well plates at the density of $3x10^5$ cells/well 24 hours before nanoparticle treatment. Each nanoparticle formulation to be tested was diluted at a concentration of 6 nM in 60% FBS / 40% PBS (phosphate-buffered saline pH 7.4) mixture and incubated overnight (14-16 hours) at room temperature to allow protein-corona formation and equilibration (**Figure S7a**). The following day, each nanoparticle solution was added to 5 volume equivalents of DMEM w/o Phenol Red, mixed by inversion and equilibrated at room temperature for at least one additional hour before using them with the cells. During this time the color of the solutions was monitored to exclude nanoparticle aggregation (the solution must remain red, see **Figure S7b**).

Afterwards, the medium was gently removed from the cells and replaced with 0.9 mL/well of the complete media containing the nanoparticles prepared as described above. Cells were therefore incubated in complete cell culture medium containing 1 nM of nanoparticles. Each formulation was tested in triplicate (3 wells).

After the required incubation period (3 or 24 hours at 37 °C), the nanoparticle containing medium was removed and cells were washed with complete cell culture medium (2×2 mL) and PBS (2×2 mL). Cells were then detached by treatment with 0.5 mL of a 5mM EDTA solution in PBS for 5-7 minutes at 37°C. The cell suspension was transferred to a mineralization glass vials and the wells were rinsed with additional 1.5ml of PBS by pipetting gently up and down and added

to the cell suspension. Cells were counted using an automated cell counter (Countess III, Invitrogen) and then pelleted by gentle centrifugation (5 min at 200×*g*). The supernatant was carefully removed leaving approximately 1 mL above the cell pellet. The content of the vials was dried in an oven overnight at 80-90 °C.

The dry residues were mineralized in 0.9 mL of a 2:1 v/v mixture of 65% $HNO_3$ and 36.5% HCl using a pressurized microwave acid digestion system from CEM (Discover® SP-D Clinical). After mineralization, samples were diluted by addition of 5 mL of milli-Q $H_2O$. Samples for Au quantification via ICP-MS were obtained by further dilution of 0.5 mL of these solutions to 12 mL with $H_2O$ containing L-cysteine and Bi which was used as an internal standard. The diluted samples contained < 2% v/v acid mixture, 0.1% w/v L-cysteine and 5.0 µg/L Bi. The gold concentration was determined by comparison with an external calibration curve (8 concentration levels in the interval 0.5 – 30.0 µg/L).

The quantification of the number of AuNP/cell after 24 h resulted in lower absolute numbers and higher data scattering (**Figure S8** left). This is attributed to the strong proliferation of this cell line under the incubation conditions in complete cell culture medium. In fact, the average number of cells increases more than 6 times during the 24-hours incubation (**Figure S8** right) with a corresponding increase of the associated standard deviation. This strong proliferation is a confounding factor for the evaluation of the nanoparticle uptake at 24 h. Microscopy images of the cell cultures after 24 hours incubation in the presence of AuNPs are shown in **Figure S7c-g**. No significant morphological variation was observed.

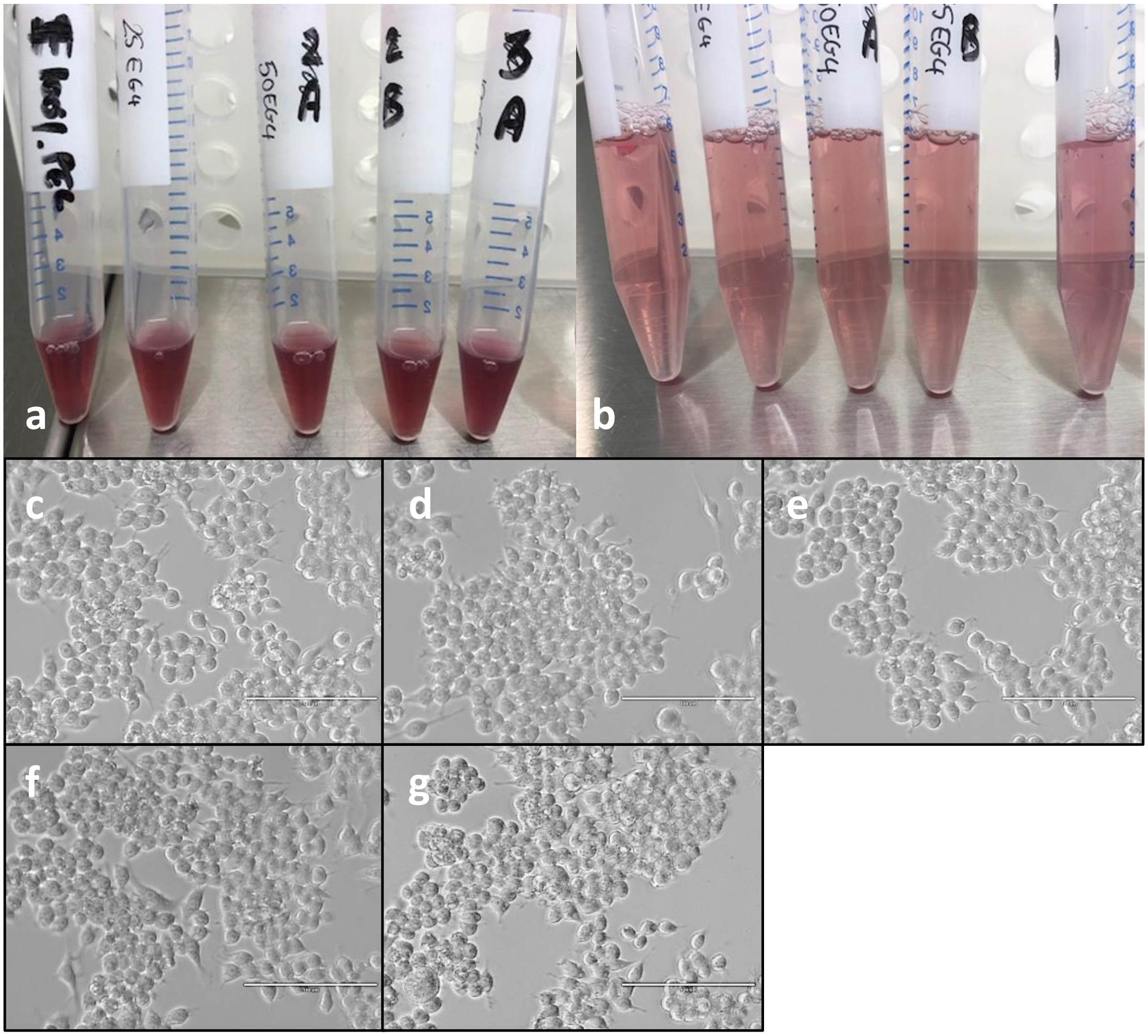


**Figure S7.** Nanoparticle internalization experiments: a) Left to right: Nanoparticle formulations ***hete*-EG$_n$**, ***mix*-25EG$_4$/PEG**, ***mix*-EG$_4$/EG$_n$**, ***mix*-75EG$_4$/PEG**, and ***homo*-EG$_4$** equilibrated overnight at r.t. in 60% FBS / 40% PBS at a nanoparticle concentration of 6.0 nM. b) Nanoparticle supplemented (1 nM) complete culture medium for internalization experiments: the previous solutions were diluted with 5 volume equivalents of DMEM w/o Phenol Red and incubated 1 h at r.t.; c-g) Murine macrophage cell line RAW264.7 after 24 h incubation with the different nanoparticle (1 nM) supplemented complete culture media: c) ***hete*-EG$_n$**, d) ***mix*-25EG$_4$/PEG**, e) ***mix*-EG$_4$/ EG$_n$**, f) ***mix*-75EG$_4$/PEG**, and g) ***homo*-EG$_4$.** Abbreviations for mixed SAMs-coated AuNPs: ***mix*-25EG$_4$/PEG**=25/75 EG$_4$/EG$_n$, ***mix*-EG$_4$/EG$_n$**=50/50 EG$_4$/EG$_n$, and ***mix*-75EG$_4$/PEG**=75/25 EG$_4$/EG$_n$. Scalebar 100 µm.

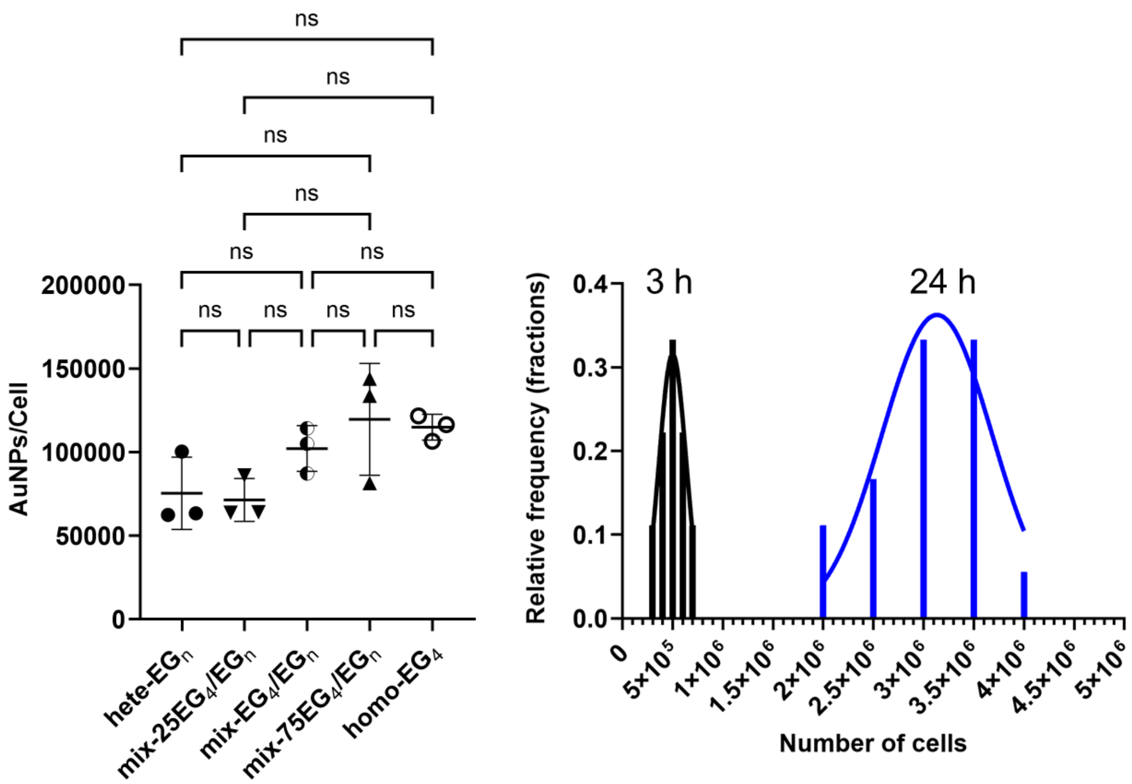


**Figure S8.** Left: Nanoparticle uptake by the murine macrophage cell line RAW264.7 after 24 h incubation with 1.0 nM of the different SAMs-coated AuNPs in complete cell culture medium, evaluated via inductively coupled plasma mass spectrometry (ICP-MS). Data are presented as scatter plots showing means ± s.d.; One-way ANOVA/Tuckey's post hoc tests. Right: frequency distributions for the number of cells incubated with 1.0 nM of the different SAMs-coated AuNPs in complete cell culture medium at 3 hours and 24 hours (distributions based on N=18 culture wells).

# References


[1] L. Maus, O. Dick, H. Bading, J. P. Spatz, R. Fiammengo, "Conjugation of Peptides to the Passivation Shell of Gold Nanoparticles for Targeting of Cell-Surface Receptors" *ACS Nano* **2010**, *4*, 6617–6628.

[2] V. Maggi, F. Bianchini, E. Portioli, S. Peppicelli, M. Lulli, D. Bani, R. Del Sole, F. Zanardi, A. Sartori, R. Fiammengo, "Gold Nanoparticles Functionalized with RGD-Semipeptides: A Simple yet Highly Effective

Targeting System for α v β 3 Integrins" *Chem. – Eur. J.* **2018**, *24*, 12093–12100.

[3] J. Hierrezuelo, J. Manuel López-Romero, R. Rico, J. Brea, M. Isabel Loza, C. Cai, M. Algarra, "Synthesis of theophylline derivatives and study of their activity as antagonists at adenosine receptors" *Bioorg. Med. Chem.* **2010**, *18*, 2081–2088.

[4] J. R. Brody, S. E. Kern, "Sodium boric acid: a Tris-free, cooler conductive medium for DNA electrophoresis" *Biotechniques* **2004**, *36*, 214–216.

[5] A. M. Davidson, M. Brust, D. L. Cooper, M. Volk, "Sensitive Analysis of Protein Adsorption to Colloidal Gold by Differential Centrifugal Sedimentation" *Anal. Chem.* **2017**, *89*, 6807–6814.

[6] Ž. Krpetić, A. M. Davidson, M. Volk, R. Lévy, M. Brust, D. L. Cooper, "High-Resolution Sizing of Monolayer-Protected Gold Clusters by Differential Centrifugal Sedimentation" *ACS Nano* **2013**, *7*, 8881–8890.

[7] A. Eliassi, H. Modarress, G. A. Mansoori, "Densities of Poly(ethylene glycol) + Water Mixtures in the 298.15−328.15 K Temperature Range" *J. Chem. Eng. Data* **1998**, *43*, 719–721.

[8] J. Austin, D. Fernandes, M. J. A. Ruszala, N. Hill, J. Corbett, "Routine, ensemble characterisation of electrophoretic mobility in high and saturated ionic dispersions" *Sci. Rep.* **2020**, *10*, 4628.

[9] A. Dukhin, S. Dukhin, P. Goetz, "Electrokinetics at High Ionic Strength and Hypothesis of the Double Layer with Zero Surface Charge" *Langmuir* **2005**, *21*, 9990–9997.

[10] C. N. Lunardi, A. J. Gomes, F. S. Rocha, J. De Tommaso, G. S. Patience, "Experimental methods in chemical engineering: Zeta potential" *Can. J. Chem. Eng.* **2021**, *99*, 627–639.

[11] P. Siani, E. Donadoni, G. Frigerio, M. D'Alessio, C. Di Valentin, "Computational Study of Ultra-Small Gold Nanoparticles with Amphiphilic Polymer Coating" *J. Compos. Sci.* **2025**, *9*, 294.

## NMR spectra

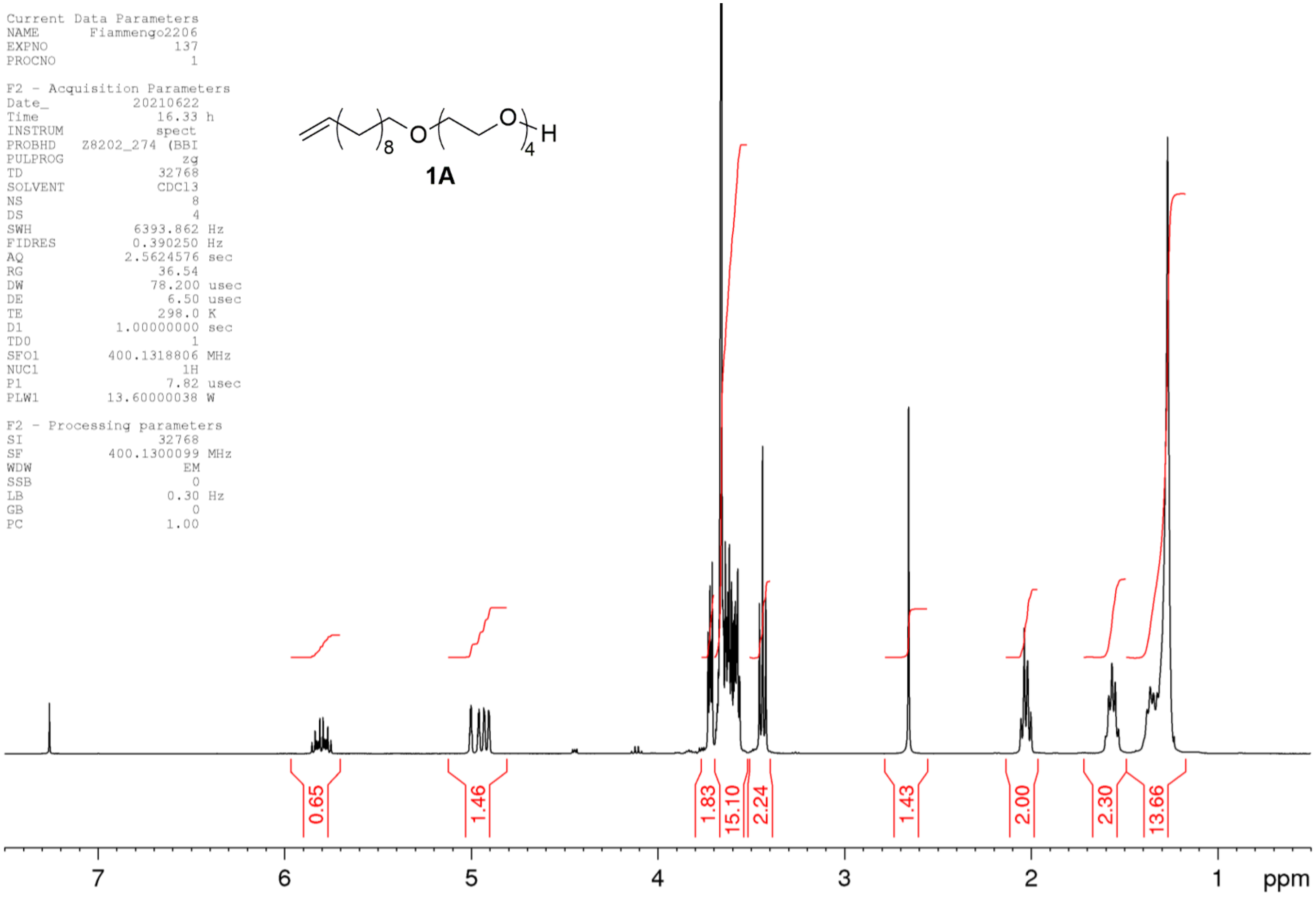


$^{1}$H NMR of compound **1A**, 400 MHz, $CDCl_3$

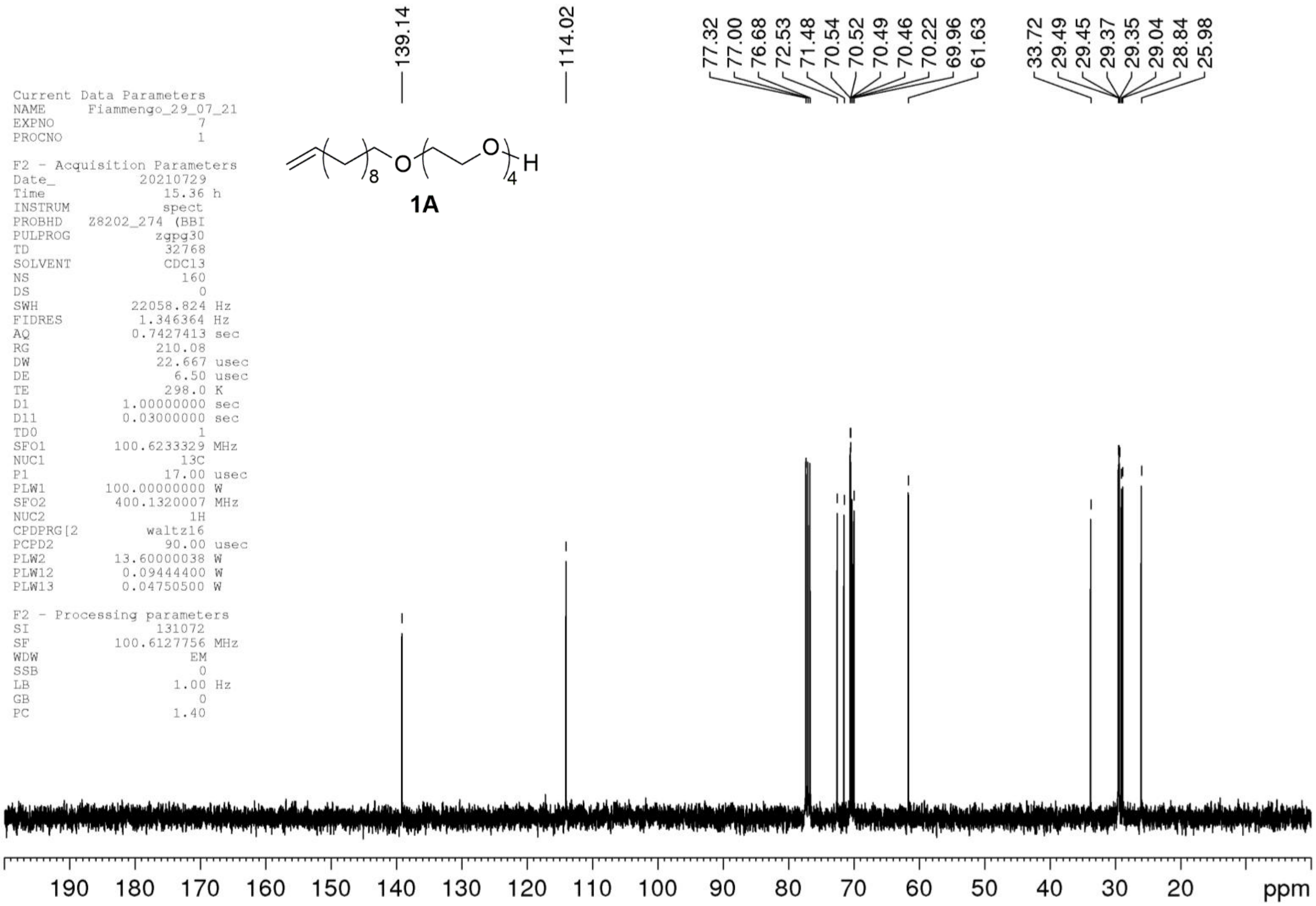


$^{13}C$ NMR of compound **1A**, 100 MHz, $CDCl_3$

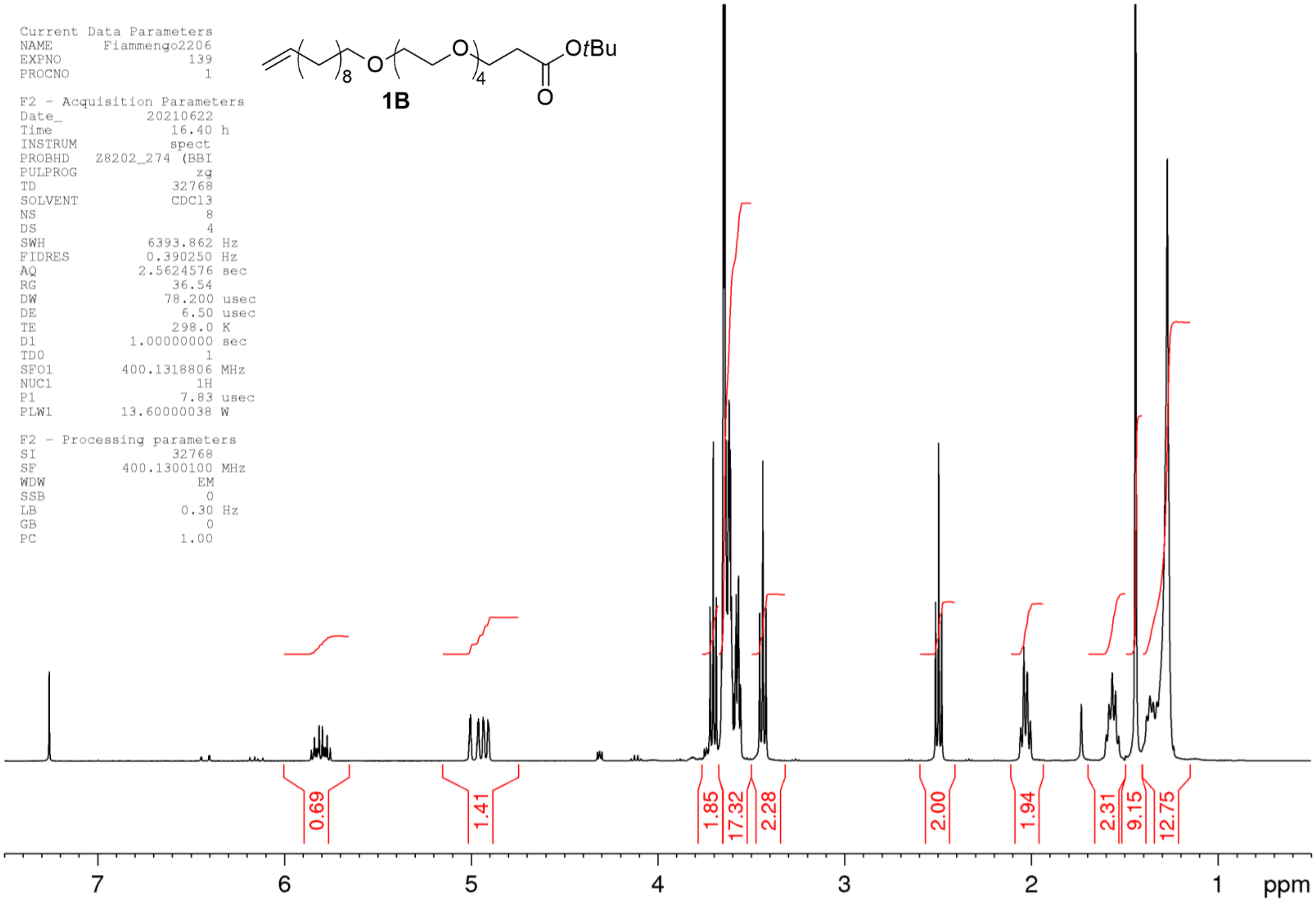


$^{1}$H NMR of compound **1B**, 400 MHz, $CDCl_3$

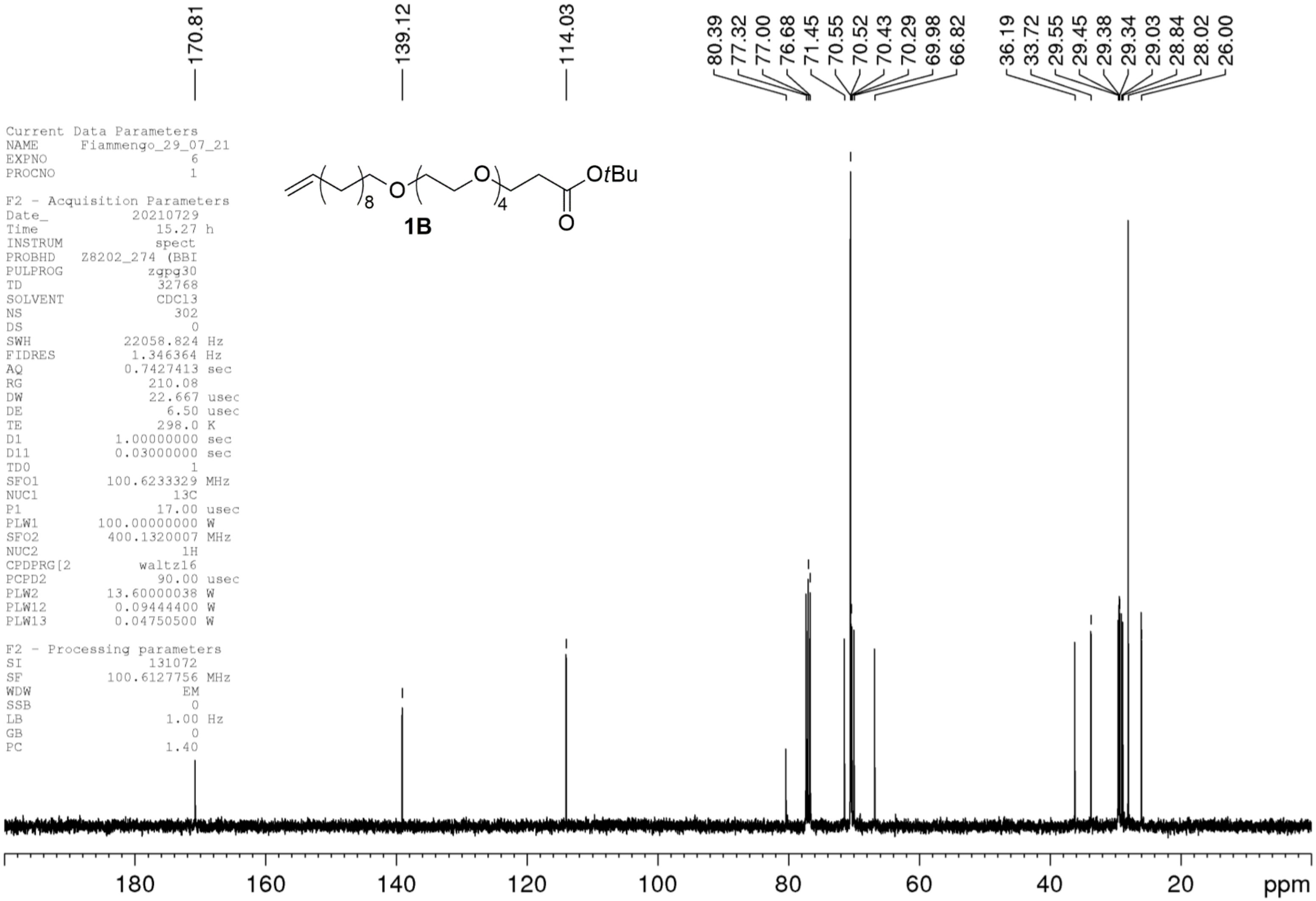


$^{13}$C NMR of compound **1B**, 100 MHz, $CDCl_3$

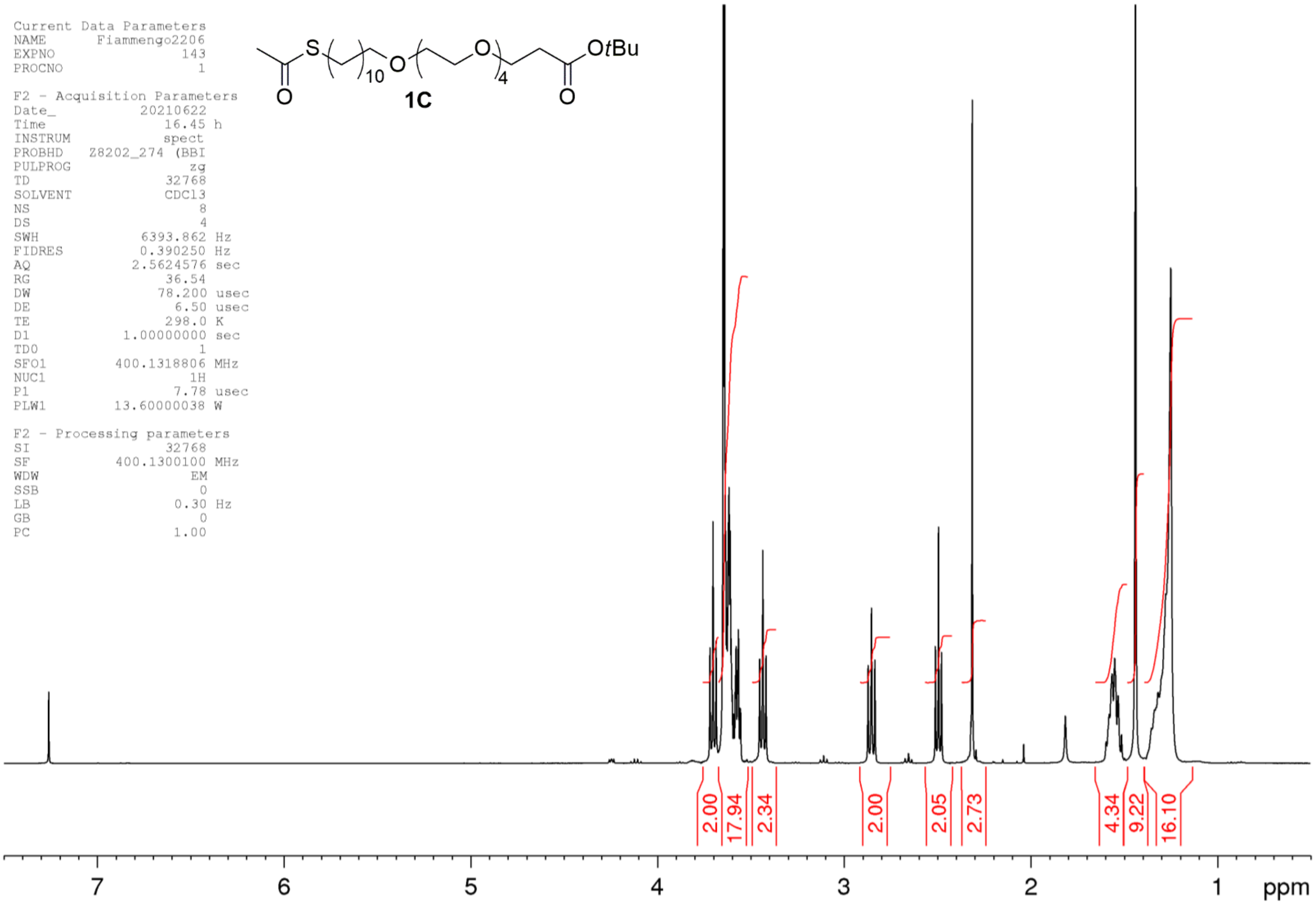


$^{1}$H NMR of compound **1C**, 400 MHz, $CDCl_3$

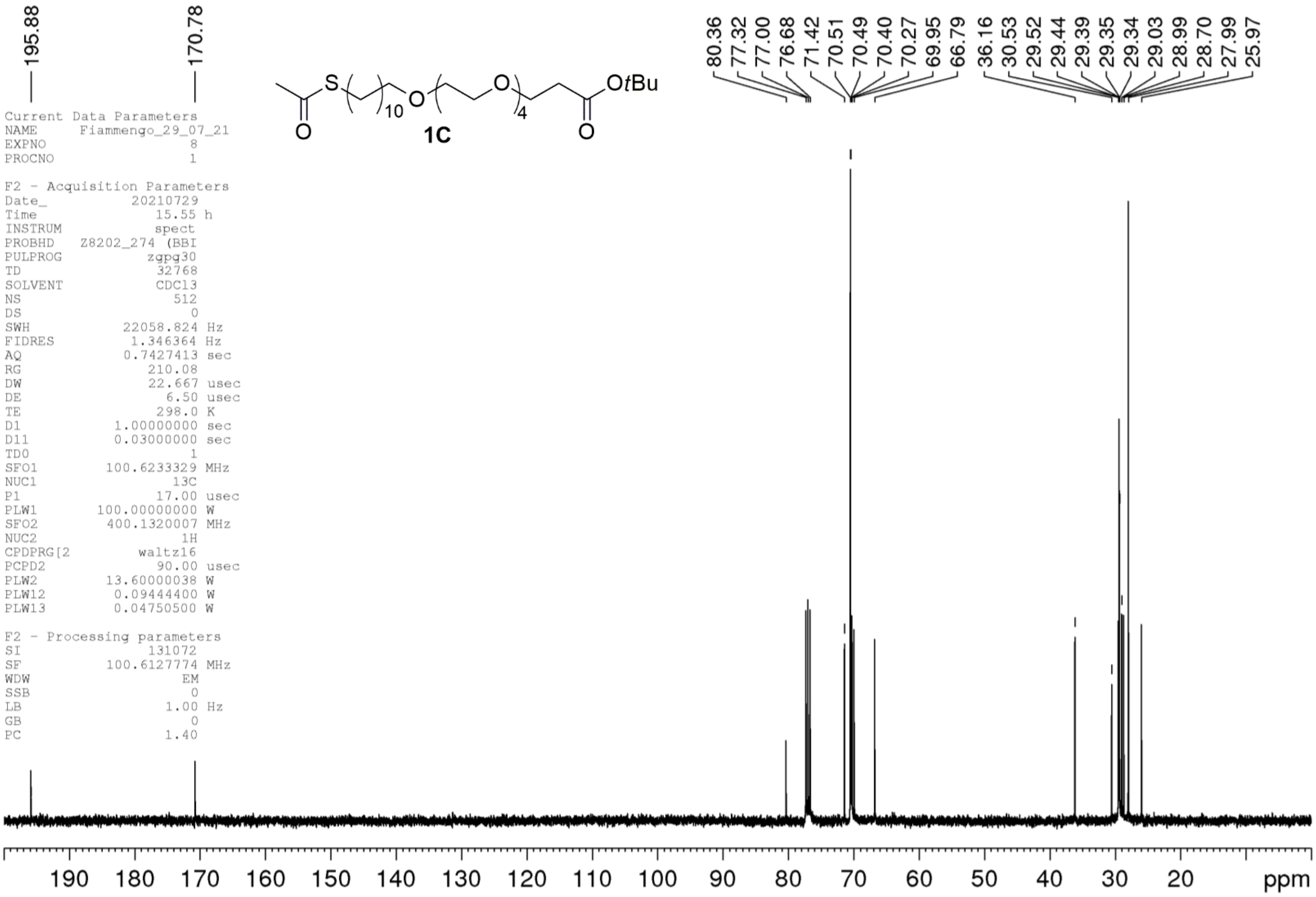


$^{13}C$ NMR of compound **1C**, 100 MHz, $CDCl_3$

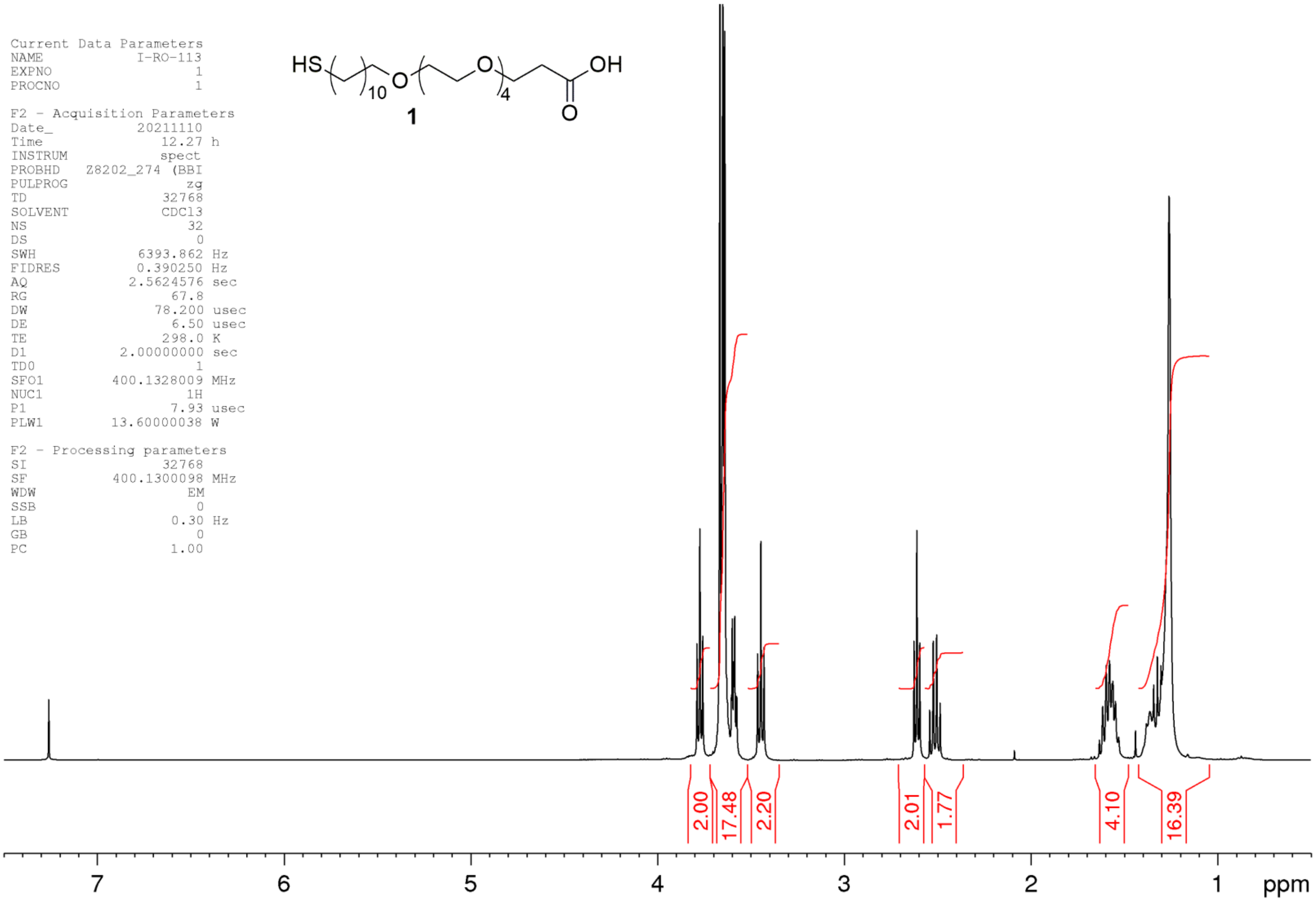


$^{1}$H NMR of compound **1**, 400 MHz, $CDCl_3$
